# Examination of oxidative stress and glutamate as potential mechanisms of N-acetylcysteine in the treatment of non-suicidal self-injury in young people assigned female at birth: randomised trial

**DOI:** 10.1192/bjo.2025.10839

**Published:** 2025-09-22

**Authors:** Victoria Papke, Bonnie Klimes-Dougan, Siddhee Anand Sahasrabudhe, Bryon A. Mueller, Young Woo Park, Gülin Öz, Lynn E. Eberly, Michaelle E. DiMaggio-Potter, Reena V. Kartha, James Cloyd, Lisa Coles, Kathryn R. Cullen

**Affiliations:** Department of Psychology, University of Minnesota, Minneapolis, USA; Center for Orphan Drug Research, Department of Experimental and Clinical Pharmacology, College of Pharmacy, University of Minnesota, Minneapolis, USA; Department of Psychiatry and Behavioral Sciences, University of Minnesota, Minneapolis, USA; Center for Magnetic Resonance Research, Department of Radiology, University of Minnesota, Minneapolis, USA; Division of Biostatistics and Health Data Science, School of Public Health, University of Minnesota, Minneapolis, USA

**Keywords:** Non-suicidal self-injury, N-acetylcysteine, randomised controlled trial, neurobiological markers, young adults

## Abstract

**Background:**

Non-suicidal self-injury (NSSI) often emerges during adolescence and young adulthood. A prior open-label pilot study suggested that N-acetylcysteine (NAC) may reduce NSSI frequency in young individuals.

**Aims:**

This study investigated potential NSSI-related biological markers for NAC in young adults with a history of NSSI using a placebo-controlled, randomised clinical trial of two NAC dosage regimens.

**Method:**

Forty-three individuals (assigned female at birth) aged 16–24 years and with a history of NSSI were randomly assigned to either low-dose NAC (3600 mg/day), high-dose NAC (5400 mg/day) or placebo treatment for 4 weeks. Participants underwent blood draws, magnetic resonance imaging with spectroscopy and clinical assessments before and after treatment. Primary outcomes included brain glutathione (GSH), blood reduced to oxidised GSH ratio and brain glutamate. Secondary outcomes included antioxidant protein levels, brain gamma-aminobutyric acid concentrations, functional connectivity (between amygdala and insula) and clinical outcomes. Pharmacokinetics, tolerability and correlations among measures were also explored.

**Results:**

For 39 participants who completed study assessments at follow-up, weekly NSSI and depression symptoms improved similarly across both treatment and placebo groups, with no significant group differences in primary or secondary outcomes at follow-up. Some significant correlations emerged.

**Conclusions:**

The study did not support the proposed biological signatures of NAC in young adults with NSSI, although exploratory findings suggested potential biological correlates of clinical improvement. Further research is necessary to explore neurobiologically based treatments for young adults with NSSI.

Non-suicidal self-injury (NSSI), the deliberate act of damaging one’s own tissues without suicidal intent, typically emerges in early to mid-adolescence^1,2^ and is associated with severe consequences, including future suicide attempts.^3,4^ The average global prevalence of NSSI engagement is 18%,^5^ with rates highest among adolescents and young adults^6^ and recent evidence suggesting an increase amid the COVID-19 pandemic,^7^ especially in females.^8^ The World Health Organization has deemed NSSI one of the top major health threats to adolescents.^9^ However, treatments for NSSI are sorely limited. The bulk of evidence favours psychotherapeutic approaches to address NSSI,^10^ especially dialectic behavioural therapy.^11^ However, there is less support for the effectiveness of psychotropic medications, despite these being commonly prescribed to young people who engage in NSSI.^12^ That said, some pharmacological approaches have been explored, particularly opioid receptor antagonists (e.g. naltrexone), which have shown promise in small studies but lack robust, replicated evidence to support their widespread clinical use.^13^ Because the time period encompassing adolescence and young adulthood is critical for brain development,^14^ it is an opportune time frame to curtail dangerous behavioural trajectories, restore healthy behaviour and prevent negative outcomes.^15^ Identification of novel, biologically informed treatments for NSSI in young people could improve health outcomes over the lifespan.

## Potential treatment for NSSI

N-acetylcysteine (NAC), a dietary supplement and prescription medication, may have potential as a treatment for NSSI. Several reviews have suggested that NAC may alleviate a range of psychiatric problems.^16,17^ We previously conducted an open-label pilot study testing oral NAC in youth with NSSI, which showed a reduction in NSSI frequency after 8 weeks of treatment.^18^ Identification of NAC’s potential biological signatures, or measures of the mechanisms underlying its clinical effects, are needed to provide the basis for biologically informed designs of optimised efficacy trials. The present study is the first to examine biological signatures of NAC as a potential treatment for NSSI.

## Identifying biological signatures

Recent efforts have promoted experimental therapeutics approaches, highlighting the importance of measuring a target biological mechanism of a disorder such as NSSI.^19–21^ Consistent with these efforts, the current work focused on two biological signatures. First, NAC has been shown to boost glutathione (GSH), the primary antioxidant in the blood^22^ and brain^23,24^ in humans. In animals, NAC was shown to prevent brain and behavioural alterations induced by the lesions resulting from diminution of oxidative stress.^25^ GSH can also be measured in its oxidised form (GSSG); specifically, measuring the ratio of reduced to oxidised GSH, or the GSH redox ratio (GSH:GSSG), provides helpful insight into the endogenous status of oxidative stress. In humans, this mechanism of action may provide a neuroprotective effect against the toxicities associated with oxidative stress, which has been implicated in a broad range of psychiatric problems.^16,26,27^ A second potential biological signature for NAC is the downregulation of excessive glutamate (Glu) transmission, which has been implicated in habit-based disorders.^28^ When NAC enters the body it is converted to its primary metabolite cysteine (CYS), which then enters glial cells and is exchanged for glutamate (Glu) via the cystine–Glu antiporter. As a maladaptive, habitual behaviour, NSSI overlaps conceptually with impulsive/compulsive disorders such as addiction and skin-picking, which NAC has been shown to alleviate.^29,30^ Previous research in adults with cocaine dependence suggested that NAC led to decreased Glu in the anterior cingulate cortex (ACC),^31^ a brain region implicated in regulation of emotion and control of impulses.^32^

## Aims

We conducted a randomised, triple-blind, placebo-controlled, 4-week course of NAC at two different doses (5400 and 3600 mg/day) and placebo (PBO) in youth assigned female at birth and with NSSI. We aimed to assess biological changes and select the optimal dose for achieving biological effects by examining dose/concentration–response relationships with respect to biological markers. Go/no-go criteria were used for pre-specified thresholds of change for the primary outcomes (all outcomes and measurements are listed in Table 1) to determine whether this study would progress to the next stage. In addition, we conducted a pharmacokinetic analysis of NAC and its metabolites, assessed tolerability and examined clinical changes (frequency of NSSI urges and NSSI episodes, depression severity, suicidal thoughts). Finally, we explored correlations between biological and clinical changes.

**Table 1:** Primary and secondary outcomes

Primary outcome	Study time of measurement	Predicted effect	Measurement
GSH concentration in ACC	Baseline and week 4	At least a 5% increase^33^ Degree of change will be NAC dose-dependent	MRS
GSH/GSSG	Baseline and week 4	At least a 50% increase^33^ Degree of change will be NAC dose-dependent	Blood samples
Glu concentrations in ACC	Baseline and week 4	At least a 5% decrease^31^ Degree of change will be NAC dose-dependent	MRS
Secondary outcome	Study time of measurement	Predicted effect	Measurement
GABA concentrations in ACC	Baseline and week 4	Degree of change will be NAC dose-dependent (greater change with higher dosage)	MRS
Pharmacokinetic analysis of NAC and its metabolites, CYS and GSH	Baseline and week 4	Formulation of NAC under study has approximately 5% bioavailability in circulating serum;^35^ changes in biological signatures would correlate with blood/plasma levels of NAC and its metabolites	Blood samples
Antioxidant protein levels (catalase and HO-1)	Baseline and week 4	Degree of change will be NAC dose-dependent (greater change with higher dosage)	Blood samples
Clinical symptoms	Baseline, weeks 1–4	NAC groups would show greater improvement in clinical symptoms^18^	Self-report: ISAS:^36^ frequency of NSSI behaviour ABUSI:^37^ frequency of NSSI urges BDI, PHQ-9:^38,39^ severity of depression BSSI:^40^ severity of suicidal thoughts
Resting-state functional connectivity in key brain regions	Baseline and week 4	NAC groups would show greater increase in functional connectivity between amygdala and insula^18^	fMRI: resting-state functional connectivity using FreeSurfer parcellation
Tolerability of NAC	Baseline, weeks 1–4	N/A	Self-reported: side-effects checklist

GSH, glutathione; ACC, anterior cingulate cortex; MRS, magnetic resonance spectroscopy; GSH:GSSG, blood reduced to oxidised glutathione ratio; Glu, glutamate; GABA, gamma-aminobutyric acid; PK, pharmacokinetics; NAC, N-acetylcysteine; CYS, cysteine; HO-1, haem oxygenase-1; ISAS, Inventory of Statements About Self-Injury; NSSI, non-suicidal self-injury; ABUSI, Alexian Brothers Urge to Self-Injure Scale; BDI, Beck Depression Inventory; PHQ-9, Patient Health Questionnaire-9; BSSI, Beck Scale for Suicide Ideation; fMRI, functional magnetic resonance imaging; N/A, not available.

## Method

### Participants and recruitment

Adolescents and young adults aged 16–24 years who were assigned female at birth and had a history of NSSI (based on self-reported responses to the Inventory of Statements About Self-Injury^36^) were invited to participate in this study. Further inclusion and exclusion criteria are reported in the previously published study protocol.^41^ Early in the recruitment phase, eligibility was modified to begin allowing tobacco use disorder, mild cannabis use disorder, mild alcohol use disorder or substance use disorder in early remission with abstinence ≥3 months. Participants were recruited through community and clinical settings of the University of Minnesota and screened using an online form. Written informed consent and assent was obtained from all participants, and from at least one parent or guardian for those under the age of 18. The University of Minnesota Institutional Review Board approved the study. The authors assert that all procedures contributing to this work comply with the ethical standards of the relevant national and institutional committees on human experimentation, and with the Helsinki Declaration of 1975 as revised in 2013. All procedures involving human subjects were approved by the University of Minnesota Advarra Institutional Review Board (STUDY00004148, site no. 00000329; Advarra no. 00036469).

### Trial registration

This trial was registered on ClinicalTrials.gov, with ID NCT04005053 (https://clinicaltrials.gov/ct2/show/NCT04005053).

### Intervention and study design

As described in Sahasrabudhe et al,^41^ at baseline, participants underwent a clinical assessment and were then randomised (1:1:1 parallel-arms design) to either high-dose oral NAC (high, 5400 mg/day), low-dose NAC (low, 3600 mg/day) or PBO, using a baseline adaptive minimisation procedure to balance the three treatment groups with respect to age (<20 versus 20+ years), Beck Depression Inventory (BDI-II) depressive scores (<29 versus 29+), NSSI severity (<1/week versus 1+/week) and medication use (current antidepressant use versus not).^41^ A complete schedule of other activities is reported in Supplementary Table 1 available at https://doi.org/10.1192/bjo.2025.10839, and specifications for the SW854 NAC product from Swanson Health Products are reported in the previously published study protocol.^41^

### Adherence and tolerability

Medication adherence was assessed by pill counts at weeks 2 and 4. Participants were considered to be adherent if they took at least 80% of their assigned doses. A daily survey about medication adherence was also used as a secondary measure of adherence.

A weekly side-effects checklist (administered from baseline to week 4) assessed for frequency (number of days per week the symptom was experienced) and severity (mild, moderate or severe). Side effects were categorised by affected body systems. Differences in side-effect frequency and severity between groups were examined. A binarised count comparing the rate of side effects (i.e. 0, no side effects; 1, 1 or more side effects) across groups by week and body system was also examined.

### Outcomes

Primary and secondary biological and clinical outcomes, as well as pharmacokinetic analysis and side-effects, were determined prior to data collection and are described in Table 1.

### Magnetic resonance data acquisition procedures

All brain scanning was completed on a 7T whole-body Siemens MAGNETOM scanner (Erlangen, Germany) using a single-channel transmit, 32-channel receive head matrix coil from Nova Medical and BaTiO_3_ dielectric padding.^42^ A T_1_-weighted MPRAGE image was acquired to position the magnetic resonance spectroscopy (MRS) voxel, and to facilitate registration of the functional magnetic resonance imaging (fMRI) data for group analyses. T_1_ parameters were: repetition time 2500 ms, echo time 2.4 ms, flip angle 5°, slice thickness 1 mm, number of slices 176, field of view 232 × 256 mm^2^ and matrix size 232 × 256. Proton spectra were acquired from the ACC using a voxel with the following dimensions: left–right, 2.4 cm; anterior–posterior, 3.0 cm; superior–inferior, 1.2 cm. An optimised semi-LASER sequence^43^ was used, with repetition time 5000 ms, echo time 5000 ms, with TR 5000 ms and 64 transients per spectrum. Voxel placement was based on anatomical landmarks. We utilised the AutoAlign^44^ feature for the follow-up scan in order to ensure consistent volume of interest positioning in the pre- and post-NAC scans. Acquisition methods and evaluation of cerebrospinal fluid (CSF) contribution to the ACC voxel followed our prior work.^45^ Functional data were acquired using the Human Connectome Project multiband echo planar imaging sequence for 7T.^46^ Whole-brain T_2_*-weighted functional volumes (87 contiguous slices, repetition time 1355 ms, echo time 18 ms, flip angle 52°, matrix 210 × 210 mm^2^, voxel size 1.5 mm isotropic, multiband factor 3, echo train length 40.6, echo spacing 0.66 ms) were obtained during rest. Participants were instructed to keep their eyes open while viewing a fixation cross (12 min) The duration and choice of fixation cross as the resting condition were selected to optimise reliability.^47,48^

### Magnetic resonance data processing and analysis

The methods used to quantify neurochemical profiles using LCModel,^49^ and to assess the reliability of metabolite concentrations based on Cramér–Rao lower bounds, were identical to those described previously.^45^ FreeSurfer^50^ was used to conduct initial processing of T_1_ imaging data and to parcellate the brain into standard regions of interest. After registering T_1_ data to the resting–state fMRI data, for each run of the resting-state scans we extracted the mean *z*-score from bilateral amygdala and insula regions of interest, calculated the correlation between amygdala and insula for each hemisphere and applied Fisher’s *R* to *Z* calculation. We then calculated the mean amygdala–insula connectivity value across runs per hemisphere.

### Bioanalysis of NAC, GSH:GSSG, total GSH and antioxidant proteins

Details of this are provided in Supplementary Material 1.

### Power and sample size

While our study design did not aim to reach statistical significance for a ‘clinically meaningful’ difference between groups, we still conducted a power analysis to be consistent with CONSORT expectations around clinical trials design prior to data collection, although we acknowledge a potential limitation in that estimates were obtained based on a study with a small sample size. Specifically, preliminary biosignature data came from an oral NAC pilot study in adults with either Parkinson’s disease (*n* = 4) or healthy controls (*n* = 3).^33^ Using variability estimates from Coles et al,^33^ which recorded a 6% group difference in brain GSH, power analyses revealed that a group size of 11 would have 80% power with *α* = 0.0167 (Bonferroni corrected for three pairwise comparisons) to show expected significant pairwise group differences (high versus low versus PBO) of 2.95% on change in brain GSH (our primary outcome measure), relative to the percentage of within-group change. The target sample size was set at 12 per group, allowing for one dropout per group. No interim analyses were planned or carried out.

### Statistical analysis

Within-person percentage changes in key biological measures were quantified and compared against the expected clinically meaningful changes listed in Table 1. Specifically, we explored the relationships among biological (GSH, GSH:GSSG, Glu, gamma-aminobutyric acid (GABA) and insula–amygdala connectivity) and clinical (BDI-II, BSSI, ABUSI, PHQ-9, ISAS, natural log (*ln* ) total NSSI episodes and injuries) changes. These were conducted for the whole sample and within each group. For clinical outcomes, change was defined as post-treatment minus baseline scores. For biological outcomes, change was defined as the percentage change between post-treatment and baseline visit data.

To examine dose-dependent effects on key measures of interest, we fit a series of general linear models with each key primary and secondary outcome measure (primary: brain GSH concentrations, GSH:GSSG concentration ratio and brain Glu concentrations; secondary: GABA concentrations, antioxidant protein level (catalase and haem oxygenase-1 (HO-1)), amygdala–insula functional connectivity and clinical symptoms (NSSI behaviour, frequency of NSSI urges, severity of depression and severity of suicidal thoughts)) as the dependent variable, with group (high versus low versus PBO) as the predictor variable of interest. Our primary analyses did not adjust for the variables that were balanced through the minimisation procedure, while a secondary analysis adjusted for these; because conclusions did not change, only the unadjusted results are shown here. For outcomes that were highly skewed, we used non-parametric tests instead to compare groups. For the primary analyses (brain GSH, blood GSH:GSSG, brain Glu), we used the standardised Wilcoxon test statistic with the Dwass, Steel, Critchlow–Fligner (DSCF) multiple comparison analysis,^34^ adjusting type I error using Tukey’s procedure for the three pairwise comparisons among the three groups. For the secondary analyses, we used Holm’s step-down Bonferroni type I error correction to adjust for both multiple comparisons among groups and multiple testing across the various secondary outcomes. We conducted sensitivity analyses for participants who had any changes to their medical status (e.g. change in their non-study medications during our study).

Pharmacokinetic analyses assessed NAC and GSH concentration–time data visualisation and calculation of maximum concentration (C_max_), partial NAC area under the curve (AUC_0–2h) and AUC_0–2h and C_max_ for GSH employing the linear trapezoidal rule. Descriptive statistics of the pharmacokinetic parameters were determined. Drug concentrations and other pharmacokinetic variables were correlated with degree of change in biological signatures.

To characterise tolerability, we quantified rates of side-effects and other adverse events and compared rates between groups using Poisson, survival or other rate-based models as appropriate for each event type.

All statistical analyses were conducted using R software for Windows (versions 4.0.5–4.2.1; https://cran.rstudio.com/). Statistical tests were two-tailed, with a significance level set at 0.05.

## Results

### Participants

**Fig. 1:**
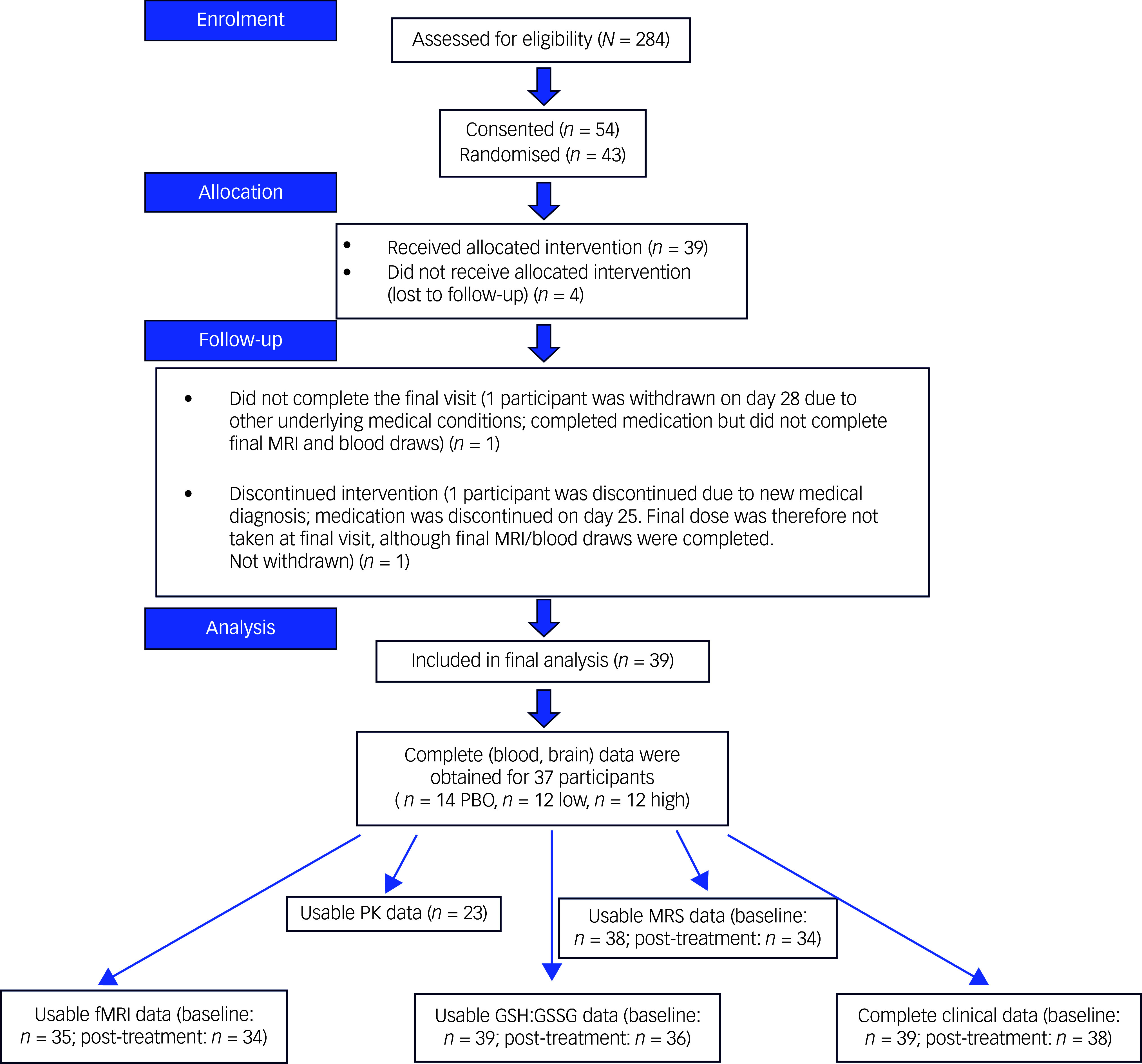
CONSORT diagram. MRI, magnetic resonance imaging; high, high-dosage N-acetylcysteine (NAC) group; low, low-dosage NAC group; PBO, placebo group; PK, pharmacokinetic; MRS, magnetic resonance spectroscopy; fMRI, functional magnetic resonance imaging; GSH:GSSG, blood reduced to oxidised glutathione (GSH) ratio.

The first participant was enrolled on 1 August 2019, and the final measures were collected from the last participant on 1 October 2022. A CONSORT diagram is provided (Fig. 1). Forty-three female young adults, aged 18–24 years and with current or recent history of NSSI, were randomised. Detailed information about their history of NSSI and STBs (including age of onset and motivations to engage in these behaviours) is shown in Supplementary Figs 1 and 2. Demographic data for the randomised participants are provided in Table 2. Complete brain and blood data were available for 37 participants (*n* = 14 PBO, *n* = 12 low, *n* = 11 high); 39 participants with partial data were included in the final analyses.

**Table 2:** Demographic and clinical characteristics of the enrolled sample

Demographics	Total (*N* = 43)	High NAC dosage (*n* = 13)	Low NAC dosage (*n* = 15)	Placebo (*n* = 15)
Age, years, mean (s.d.)	20.9 (1.91)	20.8 (1.95)	21.1 (2.00)	20.7 (1.91)
Characteristic, *n* (%)				
Assigned gender at birth				
Female	43 (100)	13 (100)	15 (100)	15 (100)
Gender identity				
Female	38 (88.37)	12 (92.3)	15 (100)	11 (73.3)
Non-binary	4 (9.30)	1 (7.7)	0 (0)	3 (20.0)
Agender	1 (2.33)	0 (0)	0 (0)	1 (6.7)
Race				
White	33 (76.74)	10 (76.9)	11 (73.3)	12 (80.0)
Asian	6 (13.95)	2 (15.4)	2 (13.3)	2 (13.3)
Black/African American	1 (2.33)	0 (0)	0 (0)	1 (6.7)
Hispanic/Latino	2 (4.65)	0 (0)	1 (6.7)	1 (6.7)
More than one race	2 (4.65)	1 (7.7)	1 (6.7)	0 (0)
Other	1 (2.33)	0 (0)	1 (6.7)	0 (0)
Income level				
Live comfortably	23 (53.49)	7 (53.8)	9 (60.0)	7 (46.7)
Meet needs with a little leftover	9 (20.93)	5 (38.5)	4 (26.7)	0 (0)
Just meet basic expenses	10 (23.26)	1 (7.7)	2 (13.3)	7 (46.7)
Don’t meet basic expenses	1 (2.33)	0 (0)	0 (0)	1 (6.7)
Clinical				
Median number of lifetime NSSI episodes (min., max.)	206 (10, 5840)	206 (14, 1240)	140 (16, 5110)	240 (10, 5840)
Psychotropic medication, *n* (%)				
Never taken antidepressant	11 (25.58)	3 (23.1)	5 (33.3)	3 (20.0)
Taken antidepressant	32 (74.42)	10 (76.9)	10 (66.7)	12 (80.0)
Clinical diagnoses, *n* (%)				
Major depressive disorder	40 (93)	13 (100)	15 (100)	12 (80)
Post-traumatic stress disorder	14 (33)	3 (23)	4 (27)	7 (47)
Generalised anxiety disorder	13 (30)	6 (46)	2 (13)	5 (33)
Panic disorder	12 (28)	3 (23)	3 (20)	6 (40)
Agoraphobia	9 (21)	2 (15)	4 (27)	3 (20)
Social anxiety disorder	9 (21)	2 (15)	2 (13)	5 (33)
Bipolar II disorder	5 (12)	1 (8)	0 (0)	4 (27)
Other	5 (12)	1 (8)	2 (13)	2 (13)
Suicidal behaviour disorder	4 (9)	1 (8)	2 (13)	1 (7)
Alcohol use disorder	4 (9)	1 (8)	0 (0)	3 (20)
Substance use disorder	3 (7)	2 (15)	0 (0)	1 (7)
Hypomanic episode	2 (5)	0 (0)	1 (7)	1 (7)
Anorexia nervosa	2 (5)	0 (0)	2 (13)	0 (0)
Binge eating disorder	2 (5)	1 (8)	1 (7)	0 (0)
Bipolar I disorder	1 (2)	0 (0)	1 (7)	0 (0)
Other specified bipolar and related disorder	1 (2)	0 (0)	1 (7)	0 (0)
Obsessive–compulsive disorder	1 (2)	0 (0)	1 (7)	0 (0)
Bulimia nervosa	1 (2)	0 (0)	1 (7)	0 (0)
Suicide attempts, *n* (%)^a^				
Never attempted suicide	24 (61.54)	8 (72.7)	9 (64.3)	7 (50.0)
Attempted suicide once	6 (15.38)	3 (27.3)	2 (14.3)	1 (7.1)
Attempted suicide twice or more	9 (23.08)	0 (0)	3 (21.4)	6 (42.9)
BDI-II score, mean (s.d.)				
Day 1	27.7 (10.1)	30.5 (10.5)	26.3 (8.74)	27.0 (11.4)
Day 28	22.8 (11.9)	26.3 (14.7)	20.6 (11.2)	22.1 (10.2)
BSSI score, mean (s.d.)				
Day 1	7.4 (6.9)	8.45 (6.2)	5.71 (7.5)	8.23 (7.0)
Day 28	6.2 (5.8)	7.20 (6.7)	3.21 (5.3)	8.36 (4.5)
ABUSI score, mean (s.d.)				
Day 1	13.4 (4.7)	14.4 (2.8)	12.9 (4.8)	13.2 (5.8)
Day 28	9.6 (4.7)	11.3 (6.1)	9.14 (3.3)	8.64 (4.7)
PHQ-9 score, mean (s.d.)				
Day 1	12.7 (4.6)	14.5 (4.6)	12.5 (4.3)	11.1 (4.7)
Day 7	10.5 (5.1)	11.5 (6.5)	10.4 (4.9)	9.77 (4.3)
Day 14	10.0 (4.8)	10.5 (5.7)	9.92 (4.5)	10.1 (4.6)
Day 21	10.1 (4.7)	11.0 (5.7)	9.36 (4.4)	10.0 (4.4)
Day 28	10.1 (4.8)	11.5 (6.2)	10.1 (4.6)	8.79 (3.6)
ISAS, ln of NSSI episodes since last visit, mean (s.d.)				
Day 1	1.4 (0.9)	1.5 (1.0)	1.4 (1.1)	1.5 (0.7)
Day 7	0.9 (0.9)	1.1 (0.9)	1.0 (1.1)	0.8 (0.8)
Day 14	0.9 (0.9)	0.9 (0.9)	0.9 (0.9)	0.8 (0.9)
Day 21	1.0 (0.8)	1.1 (0.9)	1.0 (0.8)	1.1 (0.9)
Day 28	1.0 (0.9)	0.8 (0.8)	1.1 (1.0)	0.8 (0.8)
ISAS, ln of NSSI injuries since last visit, mean (s.d.)				
Day 1	1.5 (.8)	1.5 (0.6)	1.2(1.2)	1.5 (0.9)
Day 7	1.6 (1.1)	1.7 (0.7)	1.6 (1.4)	1.7 (1.1)
Day 14	1.5 (1.0)	1.3 (0.6)	1.6 (1.4)	1.4 (1.0)
Day 21	1.4 (0.9)	1.2 (1.04)	1.3 (0.9)	1.8 (0.8)
Day 28	0.9 (.7)	1.1 (0.4)	1.0 (0.8)	1.1 (0.7)

NAC, N-acetylcysteine; ISAS, Inventory of Statements About Self-Injury; NSSI, non-suicidal self-injury; min., minimum; max., maximum; ABUSI, Alexian Brothers Urge to Self-Injure Scale; BDI, Beck Depression Inventory; PHQ-9, Patient Health Questionnaire-9; BSSI, Beck Scale for Suicide Ideation; *ln*, natural log.

a. *n* = 39.

### Adherence

Based on pill counts, the mean adherence rate of participants included in final analyses was 93% (s.d. = 0.16). The reported results include two participants (one each in the high group and the PBO group) who were classified as non-compliant (pill count of <80%), given that sensitivity analyses revealed that the results were largely similar when excluding these two participants. Results from the secondary adherence measure (daily survey) are provided in Supplementary Table 2.

### MRI results

For magnetic resonance imaging (MRI) data the quality of spectra was high, allowing for the quantification of all metabolites of interest. As shown in the CONSORT diagram, usable MRI data were available for 38 participants at baseline and 34 participants post-treatment. Out of 78 MRI sessions at which fMRI data were collected, we excluded 9: 2 because replacement data of better quality were collected during a re-scan session, 4 due to poor structural data quality and 3 due to excessive motion in the fMRI data (>40% of volumes exceeding our threshold of acceptable motion). Thus, usable fMRI data were available for 35 participants at baseline and 34 participants post-treatment.

### Clinically meaningful biological change

The only biological change that almost met *a priori* criteria for a clinically meaningful change was ACC GSH levels in the low group (increased by 4.77%; criterion was 5%; Fig. 2 and Supplementary Table 3). Pre- and post-treatment average levels of all biological outcomes by group are shown in Supplementary Figs 3–9.

**Fig. 2:**
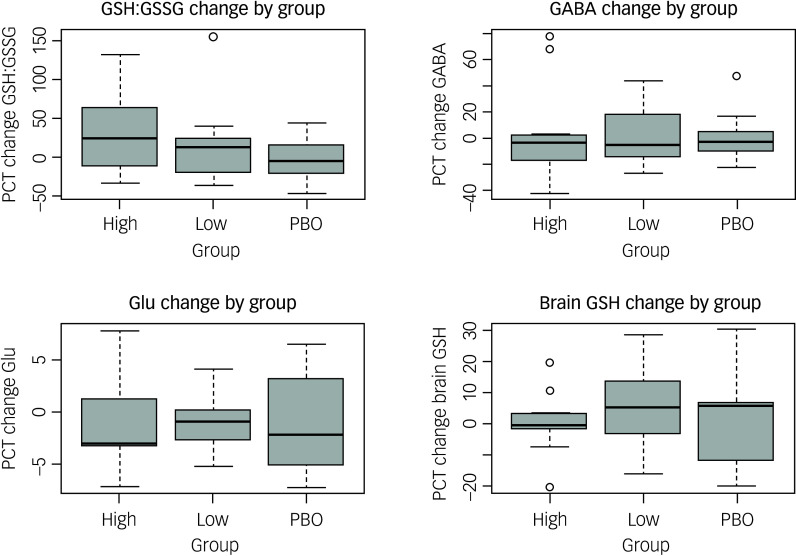
Percentage change (PCT) of key biological outcomes by treatment group. High, high-dosage N-acetylcysteine (NAC) group; low, low-dosage NAC group; PBO, placebo group; GSH:GSSG, blood reduced to oxidised glutathione (GSH) ratio; GABA, gamma-aminobutyric acid; Glu, glutamate.

### Dose-dependent biological effects

There were no statistically significant group differences in change for any key biological outcomes (Fig. 2). Means, standard deviations and 95% confidence intervals are reported in Supplementary Table 3 (absolute change and percentage change), and non-parametric statistical tests results for group comparisons for percentage change in primary and secondary biological outcomes are shown in Table 3.

**Table 3:** Group comparisons for percentage change

Parameters	High versus low	High versus PBO	Low versus PBO
**Outcome**	Wilcoxon test statistic	Wilcoxon test statistic	Wilcoxon test statistic
**Brain GSH**	**1.11**	**0.53**	**0.62**
**Brain Glu**	**0.78**	**0.53**	**0.25**
**Blood GSH:GSSG**	**1.13**	**2.25**	**1.15**
Brain GABA	0.37	0.62	0.18
Right hemisphere amygdala–insula rsFC	1.68	0.37	1.22
Left hemisphere amygdala–insula rsFC	3.17	3.36	0.24
Hemisphere-averaged amygdala–insula rsFC	4.01	4.20	0.49

High, high-dosage N-acetylcysteine (NAC) group; low, low-dosage NAC group; PBO, placebo group; GSH, glutathione; Glu, glutamate; GSH:GSSG, blood reduced to oxidised glutathione ratio, based on blood GSH and GSSG; GABA, gamma-aminobutyric acid; rsFC, resting-state functional connectivity. Non-parametric tests (banked on ranks) of percentage changes pre- and post-treatment across groups. Primary outcomes are shown in bold. *P < 0.05.

### Pharmacokinetic analysis of NAC exposure

The NAC concentration in PBO samples was below the level of quantitation for our assay. We did not observe any relationship of dose with partial AUC (Supplementary Table 4).

### Clinical outcomes

Depression and NSSI decreased in all groups, but there were no significant differences between groups in regard to the extent of change over time (see Supplementary Table 5). Pre- and post-treatment average levels of all clinical outcomes by group are shown in Supplementary Figs 10–15.

### Exploratory findings from clinical–biological change correlation analyses

Within- and whole-group heat maps showing the correlations between clinical and biological outcomes are shown in Supplementary Figs 16–19. Across the whole sample, an increase in GSH:GSSG blood ratio percentage change correlated with improvement in suicidal severity (*r*(32) = −0.42, *P* = 0.014). In the high group, improvement in suicidal severity correlated with increased Glu (*r*(8) = −0.74, *P* = 0.014). Improvement in depression PHQ-9 correlated with an increase in GSH:GSSG blood ratio (*r*(9) = −0.72, *P* = 0.013). Contrary to hypotheses, a decrease in GSH:GSSG blood ratio correlated with a decrease in NSSI injuries per week (calculated as *ln* (*r*(4) = 0.84, *P* = 0.036). In the low group, a decrease in Glu correlated with improvement in depressive symptoms (as measured by BDI-II; *r*(11) = 0.62, *P* = 0.025). An increase in GSH:GSSG blood ratio correlated with a decrease in the *ln* of NSSI episodes per week (*r*(6) = −0.78, *P* = 0.022). Lastly, a decrease in amygdala−insula connectivity correlated with the *ln* of NSSI injuries per week (*r*(5) = 0.77, *P* = 0.041). In PBO, Glu correlated with improvement in depression PHQ-9 (*r*(10) = 0.59, *P* = 0.043).

### Tolerability

During the study, one serious adverse event occurred: a participant in the PBO group was hospitalised due to suicidality. Differences in side-effect frequency and severity among groups were also examined (Supplementary Tables 6 and 7). Tachycardia was the most commonly reported cardiovascular symptom, and headaches the most commonly reported nervous system symptom. A side-effect frequency of 1–2 days and a severity of mild or moderate were most commonly reported. Several group differences emerged in the side-effect profile, with those in the high group showing more severe side-effects. Cardiovascular symptom severity at week 1 was greater in high versus low (*β* = 3.88, *P* = 0.0167) and in high versus PBO (*β* = 3.82, *P* = 0.0189); and at week 4, high versus PBO (*β* = 3.69, *P* = 0.0247). Nervous system side-effects at week 1 were greater in high than in PBO (*β* = 3.79, *P* = 0.0201), and at week 2 were greater for high versus low (*β* = 3.51, *P* = 0.0347). High showed greater symptoms than low in the ear, nose and throat category at baseline (*β* = 3.41, *P* = 0.0418). At week 1, high showed greater frequency of gastrointestinal symptoms than both low (*β* = 3.62, *P* = 0.028) and PBO (*β* = 5.32, *P* = 0.0005); this effect was also found at week 3 for high versus both low (*β* = 3.42, *P* = 0.041) and PBO (*β* = 4.01 *P* = 0.0128), and at week 4 versus PBO (*β* = 3.71, *P* = 0.024). High showed greater frequency of genito-urinary symptoms at week 1 than both low (*β* = 3.40, *P* = 0.0427) and PBO (*β* = 3.51, *P* = 0.0348). At week 4, high had greater musculoskeletal symptoms than PBO (*β* = 4.03, *P* = 0.0122). Finally, for skin-related side effects, there was a significant difference at week 1 in frequency between the high and low groups (*β* = 3.44, *P* = 0.0401).

## Discussion

Despite the clear cause for concern due to the prevalence, impairment and risk for morbidity,^4,5^ there remains a paucity of research that leverages neuroscience to inform treatments for NSSI. This clinical trial investigated candidate biological mechanisms of a 4-week course of oral NAC in female adolescents and young adults with NSSI. Key study strengths include the randomised design and the examination of brain and blood outcomes, together with pharmacokinetic analysis. The study results did not support our hypotheses regarding the extent of change in the proposed biological signatures or dose-dependent effects. However, findings emerged regarding links between changes in biological measures with those in key clinical symptoms, which may suggest potential future avenues for research.

There are two possible explanations for the lack of between-group effects on biological signatures: oral NAC has low bioavailability and it may have low penetrance into the CNS. Our findings suggest that the exposure of NAC was not correlated with administered doses, despite reports of good study medication adherence. Several studies on oral NAC have demonstrated peripheral measures of antioxidant change,^22,33^ but there is currently a paucity of evidence demonstrating that oral NAC leads to central antioxidant effects. While some reports have questioned whether NAC crosses the blood–brain barrier,^35,51^ two studies showed that oral NAC leads to dose-dependent NAC levels in CSF^52,53^ and, based on animal models, oral doses of 35 and 70 mg/kg led to NAC CSF levels surpassing those known to have significant antioxidant effects on neurons.^53^ However, prior research has shown that intravenous NAC has stronger biological effects than oral NAC.^54^ In a small study of patients with Parkinson’s or Gaucher’s disease, a single intravenous dose of NAC led to acute, immediate increases in GSH redox ratio in blood (200-fold), and in cortical GSH concentrations (30–50%) as measured by MRS.^23^ However, following a 28-day course of high-dose oral NAC (6000 mg/day), the antioxidant effects in both blood and brain were more modest and statistically insignificant (6% increase in brain GSH, twofold increase in blood GSH/GSSG).^33^ The current findings were more in line with the latter study; with our larger and younger sample, the changes demonstrated were smaller in both measures. Importantly, the goals of this study were not to demonstrate statistical significance, but rather to demonstrate changes meeting the thresholds defined *a priori* for each biomarker, constituting the go/no-go criteria for advancing to the next phase of the study, which would be a randomised controlled trial designed to have high statistical power to detect a clinically meaningful group difference. Future studies investigating NAC mechanisms may benefit from other modalities of drug delivery, to achieve better bioavailability and impact on both central and peripheral biological markers.

Lack of support for the proposed biological signatures may also have been related to power. While our study’s power analysis was based on data from prior research examining the impact of NAC on brain and blood measures of GSH in adults with neurodegenerative disorders,^33^ it may be that there is greater biological heterogeneity in individuals who engage in NSSI, thus requiring larger samples. Variance could arise from multiple sources, including variance in the co-occurring psychiatric diagnoses, presence versus absence of past adverse experiences or varying motivations for NSSI (e.g. to regulate negative affect versus socially motivated NSSI). Ideally, randomisation would address these concerns but the small sample may not optimally account for these potential confounds. In line with this thinking, using a two-sample *t*-test with Bonferroni corrections for pairwise comparisons, post hoc power calculations revealed that for 80% power to detect a PBO versus high group difference of the same magnitude that we observed, future studies should include substantially larger sample sizes (e.g. *N* = 98 per group).

One strength of this study was the inclusion of a placebo comparison group, which is imperative for distinguishing treatment-specific effects on changes from other factors, such as the placebo effect. Notably, some biological changes were observed in the PBO group, suggesting that changes in oxidative state across all treatment groups may, in part, be due to placebo effects. This effect has been similarly reported in other studies (e.g. ref. ^55^).

Although not powered as an efficacy trial, we still explored group differences in clinical improvement. In keeping with a previous open-label study demonstrating reductions in depression severity and NSSI following 8 weeks of oral NAC in youth with NSSI,^18^ we again showed similar trends in clinical improvement, but, because all three groups showed a trend for decreases in NSSI frequency and depression, attribution of these changes to NAC (versus non-specific effects of being in the clinical trial) could not be confirmed. Furthermore, exploratory analyses suggested that increases in GSH correlated with changes in suicidality in the whole group, and with both depressive symptoms and suicidality in the high group.

### Limitations

Our study has several limitations that should be acknowledged. First, as previously noted, the small sample size was selected *a priori* to be capable of showing a change in brain GSH, based on prior work in a small sample of adults with neurodegenerative disorders.^33^ Sample variability in these measures may be greater in young adults with NSSI. Post hoc power analyses suggested that larger samples will be needed to show effects in future studies on young adults with NSSI; this may have particularly hindered our ability to detect group differences in fMRI. Recent developments in the field, and emerging literature, suggest that substantially larger sample sizes are required for broader neuroimaging studies to effectively detect such differences (e.g. ref. ^56^), which may be a pertinent consideration for fMRI research. Second, there were several considerations pertaining to our study sample that impact generalisability. This sample was predominantly composed of White individuals who were assigned female at birth and who reported higher income. Additionally, despite efforts to recruit participants between the ages of 16 and 24 years, the age range of participants ultimately enrolled was 18–24 years. While we intentionally chose this age range and focused on females due to their higher prevalence of NSSI, the characteristics of our sample may have limited the generalisability of our results to other demographic groups. It is currently unknown how the biological impacts of NAC may vary across demographic groups; this represents a potentially important area of future study. Furthermore, most of the participants in this study met criteria for major depressive disorder. The biological signatures of NAC may vary across different psychiatric conditions other than depression. Moreover, many of our participants were currently taking psychotropic medication, which may represent an additional factor impacting the biological effects of NAC.

Although findings did not support the hypothesised biological changes, we observed some potential trends suggesting biological correlates of clinical improvement. Future research investigating NAC as a potential intervention for NSSI may benefit from utilising drug delivery methods capable of ensuring greater bioavailability and from using larger samples in anticipation of biological variability.

## Supporting information

Papke et al. supplementary material Papke et al. supplementary material

## Data Availability

The data that support the findings of this study are available from the corresponding author, K.R.C., on reasonable request.

## Supplementary material

The supplementary material is available online at https://doi.org/10.1192/bjo.2025.10839
