# Supplementary material for: Examination of oxidative stress and glutamate as potential mechanisms of N-acetylcysteine in the treatment of non-suicidal self-injury in young people assigned female at birth: randomised trial
Source: BJPsych Open. 2025 Sep 22;11(5):e221. doi: 10.1192/bjo.2025.10839 (PMC12458090; doi:10.1192/bjo.2025.10839)
Supplement: Papke et al. supplementary material [file S2056472425108399sup001.docx]

**Supplementary Materials**

**Table S1.** Schedule of events and assessments.

| **Measure/Activity/Event** | **Baseline (Zoom)** | **Baseline (In-Person)** | **Week 1 (Zoom)** | **Week 2 (Zoom)** | **Week 3 (Zoom)** | **Week 4 (In-Person)** |
| --- | --- | --- | --- | --- | --- | --- |
| Informed Consent Form (HIPAA/Consent/Assent) | × |  |  |  |  |  |
| Mini International Neuropsychiatric Interview (MINI) | × |  |  |  |  |  |
| Wechsler Abbreviated Scale for Intelligence-II  (WASI-II) |  | × |  |  |  |  |
| Edinburgh Handedness Inventory | × |  |  |  |  |  |
| Demographics Form | × |  |  |  |  |  |
| Childhood Trauma Questionnaire (CTQ) | × |  |  |  |  |  |
| Antidepressant Medications | × |  |  |  |  |  |
| Any Psychotropic Medications | × |  |  |  |  |  |
| Treatment History | × |  |  |  |  |  |
| Inventory of Statements About Self-Injury-Lifetime (ISAS-Lifetime) | × |  |  |  |  |  |
| Distress Tolerance Scale (DTS) | × |  |  |  |  |  |
| Beck Scale for Suicidal Ideation (BSS) |  | × |  |  |  | × |
| Patient Health Questionnaire (PHQ-9) |  | × | × | × | × | × |
| Ongoing Medication Use and Changes: Initial Visit |  | × |  |  |  |  |
| Ongoing Medication Use and Changes: Subsequent Visit |  |  | × | × | × | × |
| Alexian Brothers Urge to Self-Injure (ABUSI) |  | × |  |  |  | × |
| Beck Depression Inventory (BDI-II) | × |  |  |  |  | × |
| Self-Injurious Thoughts and Behaviors Interview (SITBI) | × |  |  |  |  | × |
| Inventory of Statements About Self-Injury-Since Last Visit (ISAS-SLV) |  | × | × | × | × | × |
| Deliberate Self-Harm Questionnaire, Part III Mood (DSHQ-M) |  | × |  |  |  | × |
| Cash Choice Task |  | × |  |  |  | × |
| Medication Side Effect Checklist |  | × | × | × | × | × |
| Magnetic Resonance (MR) Safety Screen, Urine Toxicology Screen, Pregnancy Test |  | × |  |  |  | × |
| MRI, MRS, rs-fMRI, Blood Sample (biomarkers) |  | × |  |  |  | × |
| Serial blood samples (PK) |  |  |  |  |  | × |

**Supplemental 1. Additional Methods Details**

### *Bioanalysis of NAC, GSH/GSSG, Total GSH and Antioxidant proteins.*

NAC and GSH concentration-time data were analyzed by non-compartmental methods. GSH/GSSG and total GSH were measured in red blood cells using high-performance liquid chromatography coupled to a tandem mass spectrometer (HPLC-MS/MS) as previously reported [[1, 2]](https://paperpile.com/c/U88ojx/ZsF1U+q9b6g). Total NAC (reduced + oxidized) concentrations were measured in plasma using a validated HPLC-MS/MS assay, with modifications to the assay as reported by King et al [[3]](https://paperpile.com/c/U88ojx/LbQmj). Briefly, a 25μl plasma sample along with the internal standard (NAC-D3, 25μl at 5μg/ml) was treated with a reducing reagent (5μl, 50mMol TCEP) for 30 min. Following this, a spontaneous derivatization reaction was carried out in the presence of 2-chloro-1-methyl pyridinium iodide (CMPI, 5μl, at 60mM CMPI, with 50μl of ammonium bicarbonate at 100mMol, incubated at room temperature for 10 minutes) which produced NAC-CMPI adduct. Following protein precipitation, and centrifugation 50μl of supernatant was diluted to 250μl with the mobile phase and the transition of the adduct (m/z= 255) to the daughter fragment (m/z=126) was tracked in the positive ionization mode.

Calibration curves were constructed for each analyte in plasma and blood using peak area response ratios of the analyte to the internal standard and nominal concentration. Concentrations of NAC, GSH/GSSG, and tGSH were calculated from the appropriate calibration curve. The lower limit of quantitation (LLOQ) was 25ng/mL for NAC and 0.5μg/mL for GSH and GSSG.

### *Determination of Catalase Activity in Red Blood Cells*

The catalase activity was assessed using the peroxidatic function of this enzyme when it

reacts with methanol in the presence of hydrogen peroxide (H_2_O_2_). The formaldehyde formed is quantified by the oxidation of chromogen, catalase purple [[4, 5]](https://paperpile.com/c/U88ojx/Sjgii+CcjgC). This assay was performed using a commercially available kit from Cayman Chemical Company (Ann Arbor, MI, USA). Briefly, RBC lysates were prepared in four times the volume of ice-cold water and then centrifuged at 10,000g for 15 min at 4°C [[6]](https://paperpile.com/c/U88ojx/HsN3t). The supernatant was collected and frozen at -80°C. For the assay, the diluted lysates (1:5,000) were incubated with methanol and H_2_O_2_ for 20 min at room temperature. The reaction was stopped by adding catalase potassium hydroxide and catalase purple. After 10 min, catalase potassium periodate was added for 5 min and absorbance was analyzed at 540 nm in a microplate reader. The values were normalized to protein concentrations determined by the Bradford method by using the Quick Start™ Bradford Protein Assay (Bio-Rad Laboratories, Hercules, CA. USA).

As a secondary measure of medication adherence, participants were sent a daily survey inquiring whether they took their scheduled medication doses on that day. **​​**Results from the daily medication tracking surveys are shown in Supplemental Table S1. Out of the 56 doses (2 per day for 28 days) that participants were asked to track via daily medication tracking surveys, they missed on average 11.6 doses (21%; standard deviation = 15.1). The average number of “Yes” responses (meaning they took their dose) was 41.8 (standard deviation = 14.6) and the average number of “No” responses was 2.6 (standard deviation = 3.1), suggesting a slight tendency to under-report missed doses.

*Determination of Plasma HO-1 Concentrations*

Determination of Plasma HO-1 Concentrations: The plasma HO-1 concentrations were determined using the commercial HO-1 ELISA kit (Enzo Life Sciences, MI, USA) following the manufacturer’s instructions with minor modification [[1]](https://paperpile.com/c/U88ojx/ZsF1U). This assay is specific for human HO-1 and does not detect the other human heme oxygenase isoforms, HO-2, and HO-3. HO-1 concentrations from the samples and the standard curve were analyzed at 450 nm in a Synergy TM 2 microplate reader and Gen 5 1.1 software (BioTek Instruments Inc., Winooski, VT, USA). All the samples were measured in duplicates. The data were analyzed by using a linear regression curve fit to calculate the plasma HO-1 concentrations for each sample, and the values are presented in ng/mL

**Table S2. Medication adherence based on self-report of *N* = 39.**

| Taken AM pill | N (%) |
| --- | --- |
| No | 61 (5.6%) |
| Yes | 805 (74%) |
| Missing | 226 (21%) |
| Taken PM pill |  |
| No | 42 (3.8%) |
| Yes | 824 (75%) |
| Missing | 226 (21%) |

**Figure S1. Age of Onset for NSSI of the Sample (based on N = 43)**


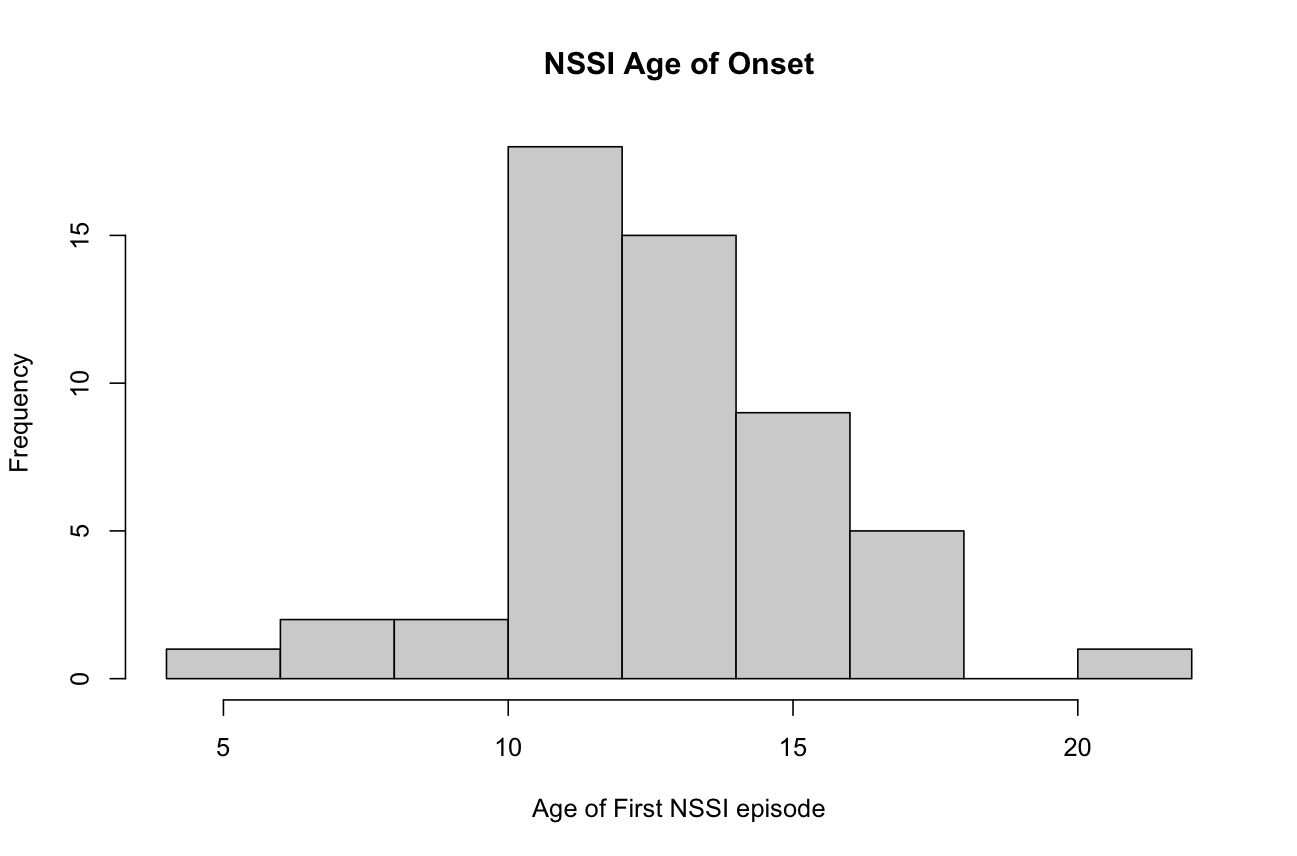


**Figure S2. Motivations for NSSI in this Sample**

**
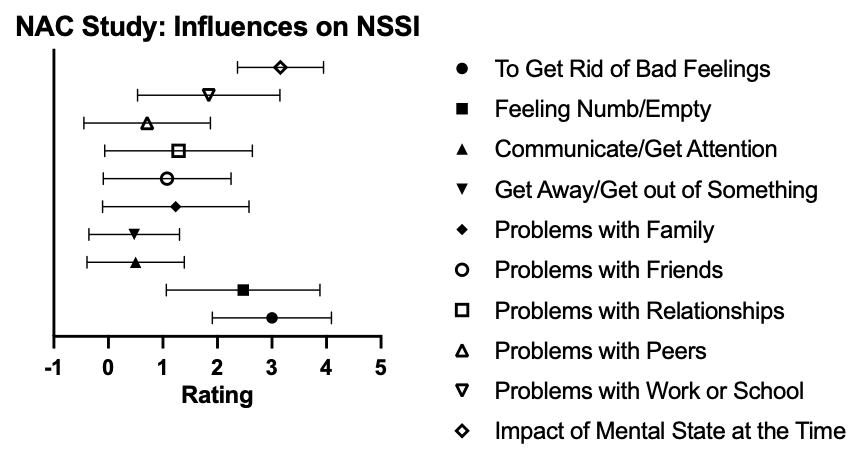
**

*Notes.* Mean responses, with standard errors, for each item. Responses were indicated on a Likert scale ranging from 0 (Low/Little) to 4 (Very Much/Severe).

**Table S3.** Change in Metabolite Concentrations Across Groups

| **Treatment Group** | **Outcome** | **Baseline Mean (SD)** | **Post Mean (SD)** | **Mean Change (95% CI)** | **Mean % Change (95% CI)** |
| --- | --- | --- | --- | --- | --- |
| Placebo | GABA | 2.18 (.37) | 2.14 (.30) | -0.04 (-0.32, 0.24) | -0.13 (-14.67, 14.42) |
|  | Gln | 2.60 (.71) | 2.86 (.59) | 0.26 (-0.039, 0.55) | 13.38 (0.85, 25.92) |
|  | Brain GSH | 1.34 (.15) | 1.37 (.19) | 0.03 (-0.07, 0.12) | 2.33 (-4.62, 9.28) |
|  | Brain Glu | 10.02 (.65) | 9.92 (.46) | -0.10 (-0.31, 0.12) | -0.76 (-2.95, 1.43) |
|  | Blood GSH/GSSG | 13.94 (4.26) | 12.86 (4.48) | -1.14 (-4.0, 1.68) | -4.68 (-29.18, 19.83) |
| Low NAC dosage | GABA | 2.08 (.44) | 2.14 (.35) | -0.00 (-0.28, 0.27) | 2.08 (-12.47, 16.62) |
|  | Gln | 2.81 (.58) | 2.69 (.70) | -0.13 (-0.42, 0.17) | -3.85 (-16.38, 8.69) |
|  | Brain GSH | 1.32 (.14) | 1.38 (.09) | 0.05 (-0.04, 0.14) | 4.77 (-2.18, 11.72) |
|  | Brain Glu | 9.98 (.39) | 9.94 (.35) | -0.10 (-0.32, 0.12) | -0.95 (-3.13, 1.24) |
|  | Blood GSH/GSSG | 14.66 (7.62) | 14.39 (5.09) | -0.55 (-3.49, 2.38) | 13.81 (-11.69, 39.32) |
| High NAC dosage | GABA | 2.21 (.62) | 2.15 (.52) | -0.06 (-0.36, 0.24) | 3.53 (-12.28, 19.34) |
|  | Gln | 2.56 (.47) | 2.30 (.51) | -0.26 (-0.58, 0.07) | -7.36 (-20.99, 6.27) |
|  | Brain GSH | 1.38 (.08) | 1.38 (.13) | 0.00 (-0.10, 0.10) | 0.41 (-7.14, 7.97) |
|  | Brain Glu | 9.81 (.47) | 9.69 (.63) | -0.12 (-0.36, 0.12) | -1.24 (-3.61, 1.14) |
|  | Blood GSH/GSSG | 12.39 (4.54) | 14.77 (4.79) | 2.39 (-0.68, 5.45) | 28.36 (1.72, 55.00) |

*Abbreviations.* GABA: brain gamma-aminobutyric acid. Gln: Glutamine. GSH: Glutathione. Glu: Glutamate. GSH/GSSG: reduced to oxidized glutathione ratio.

**Figure S3. GABA**

**
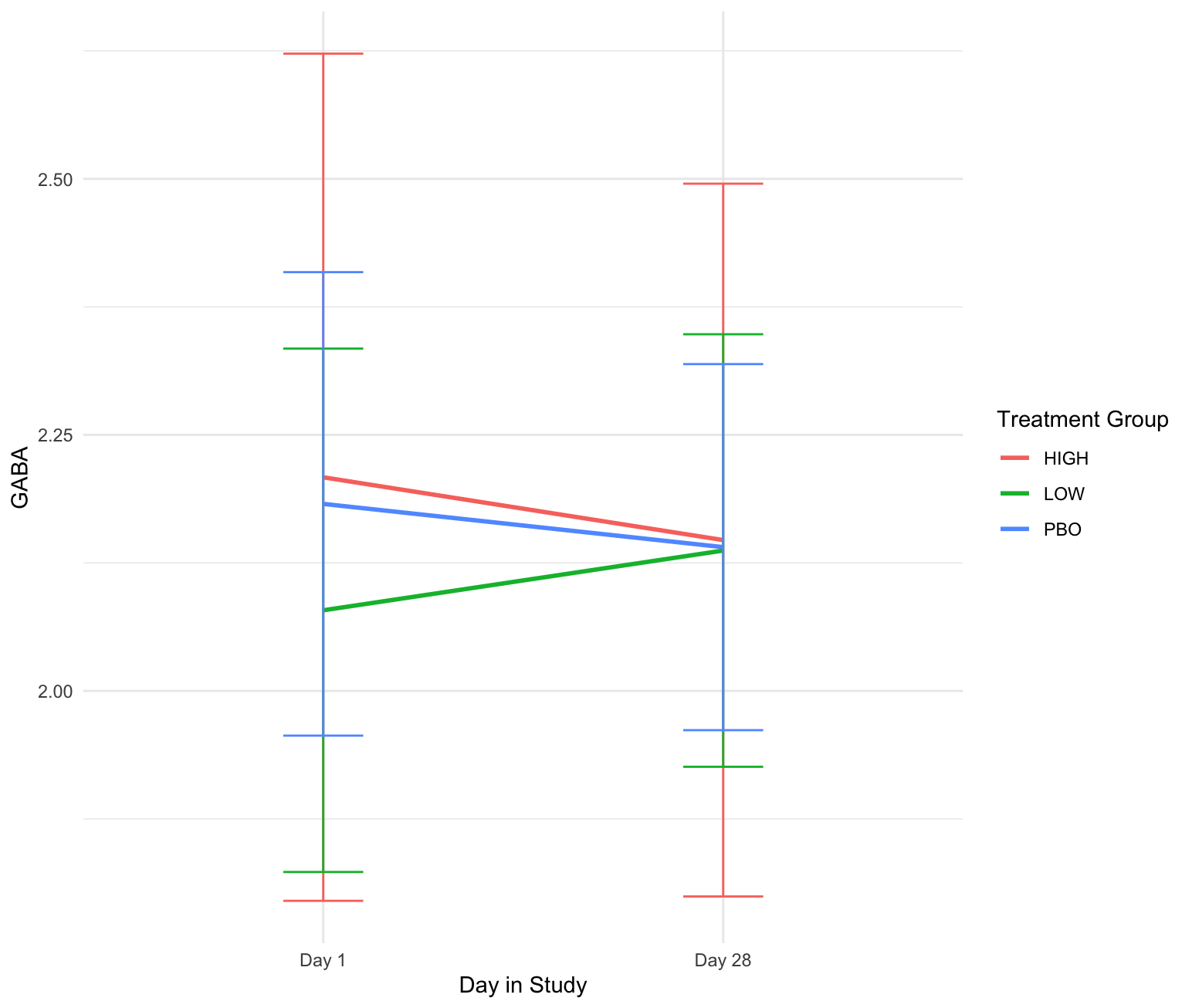
**

*Note:* Mean GABA with standard error bars across study days, by treatment group.

**Figure S4. Brain GSH**

**
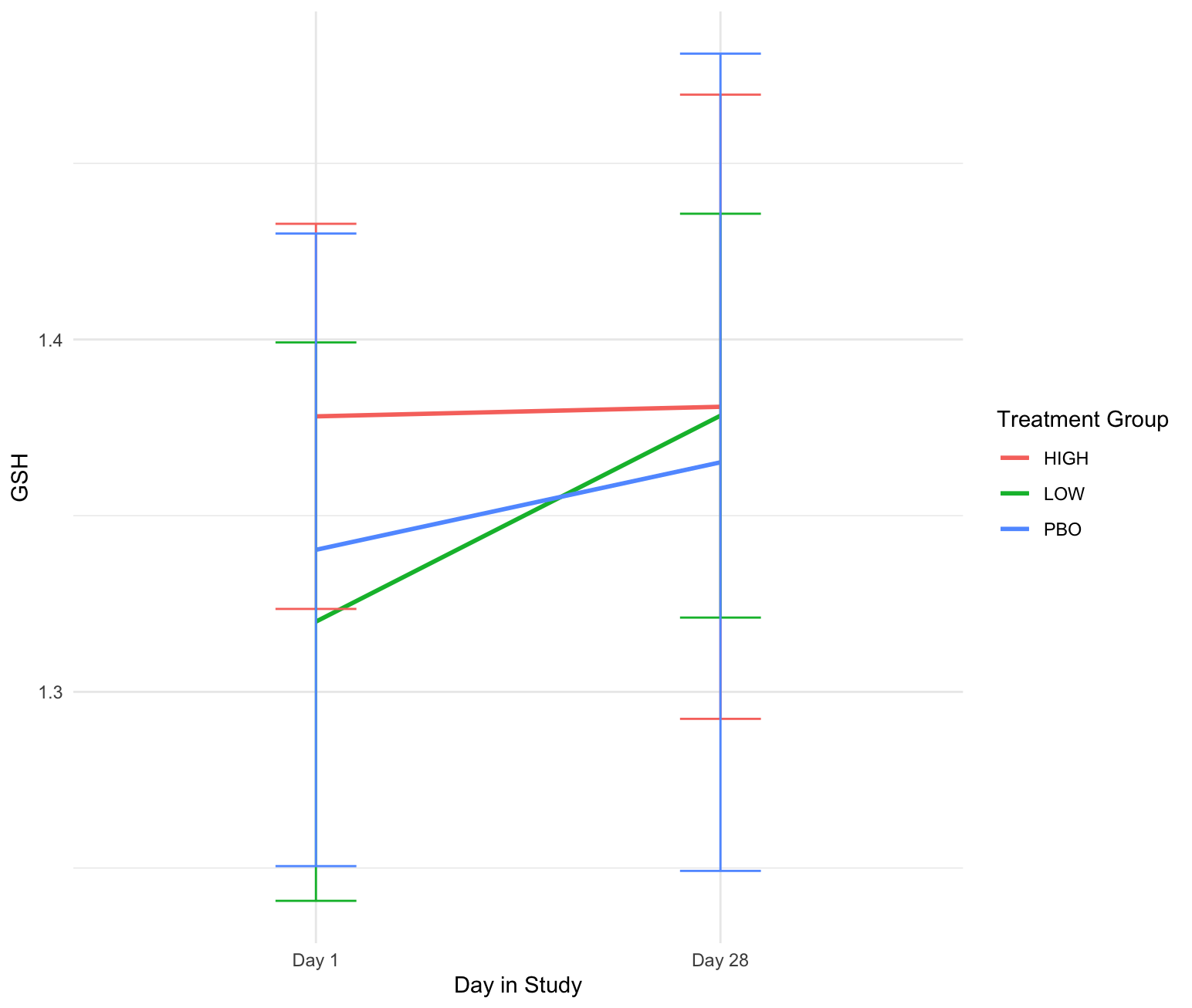
**

*Note:* Mean Brain GSH with standard error bars across study days, by treatment group.

**Figure S5. Brain Glu**

**
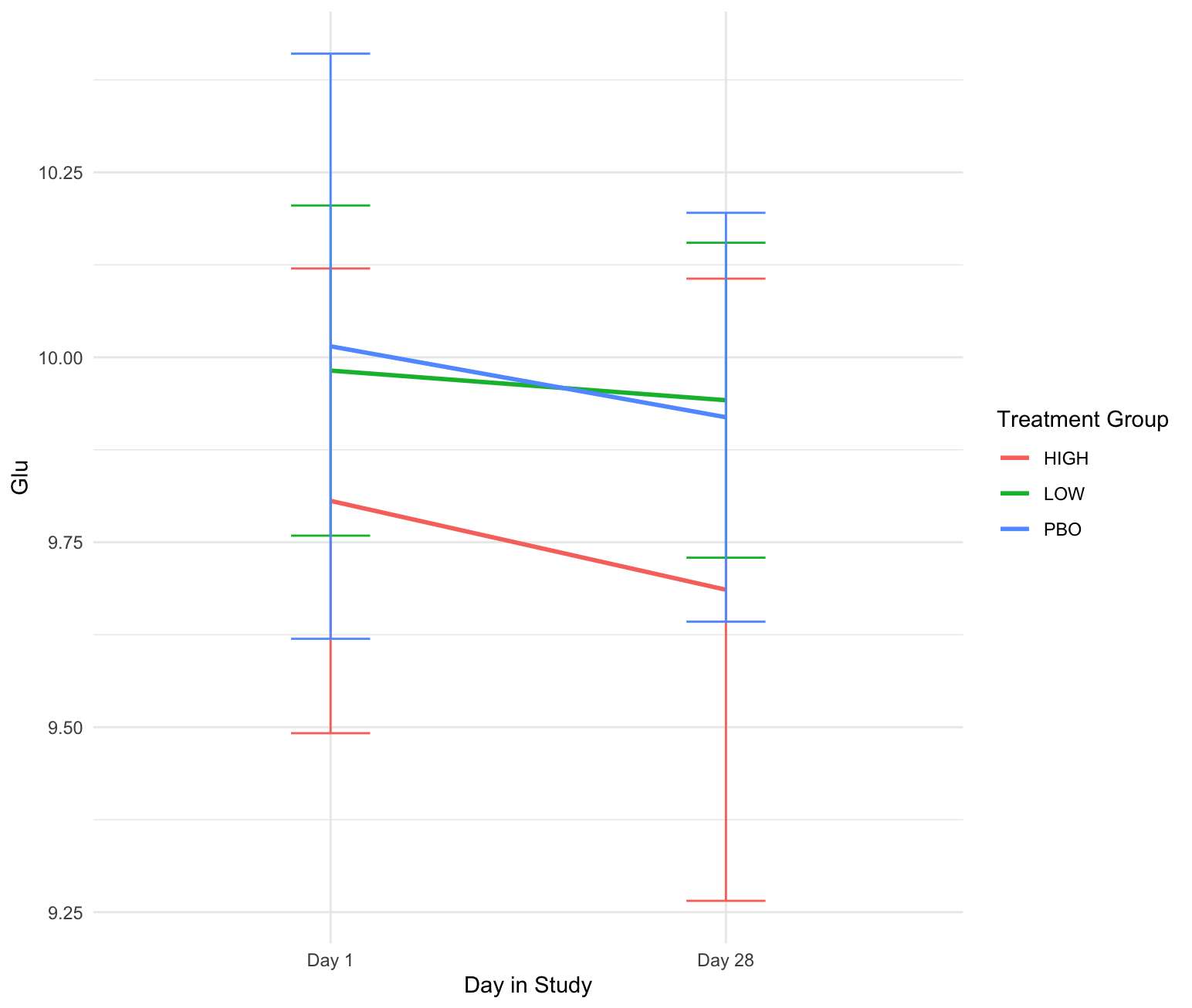
**

*Note:* Mean Brain Glu with standard error bars across study days, by treatment group.

**Figure S6. Blood GSH/GSSG Ratio**

**
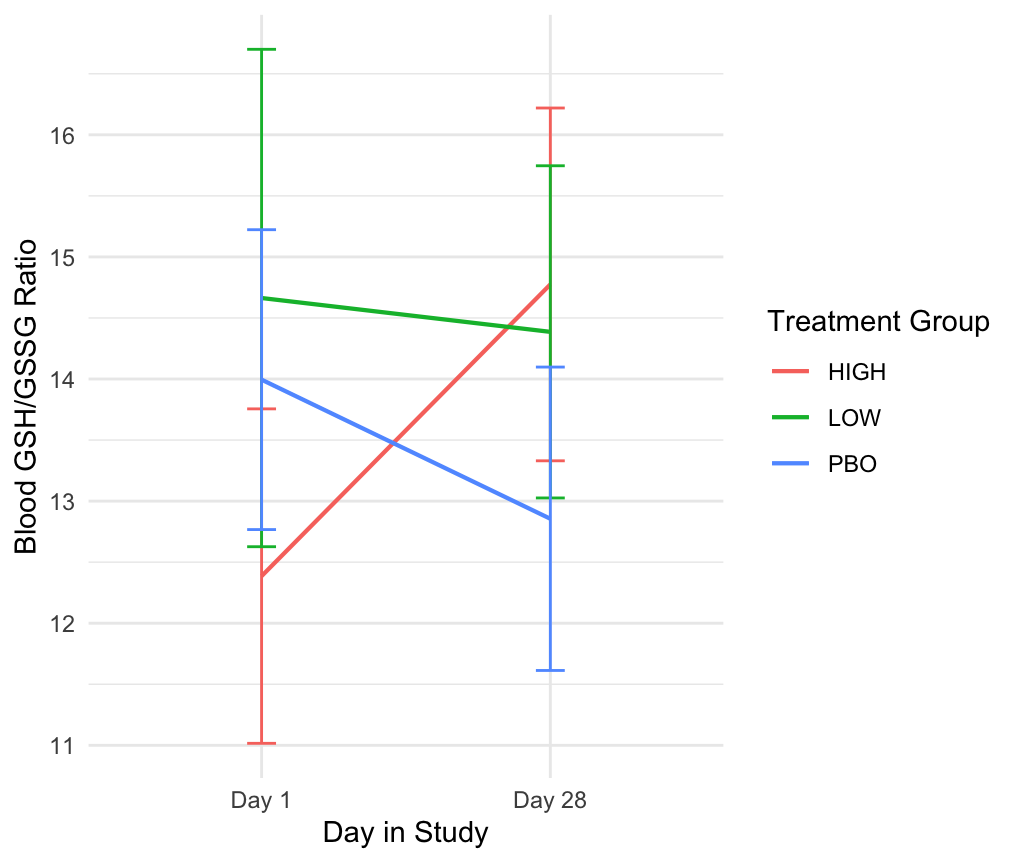
**

*Note:* Mean Blood GSH/GSSG with standard error bars across study days, by treatment group.

**Figure S7. Mean Left Insula Amygdala**

**
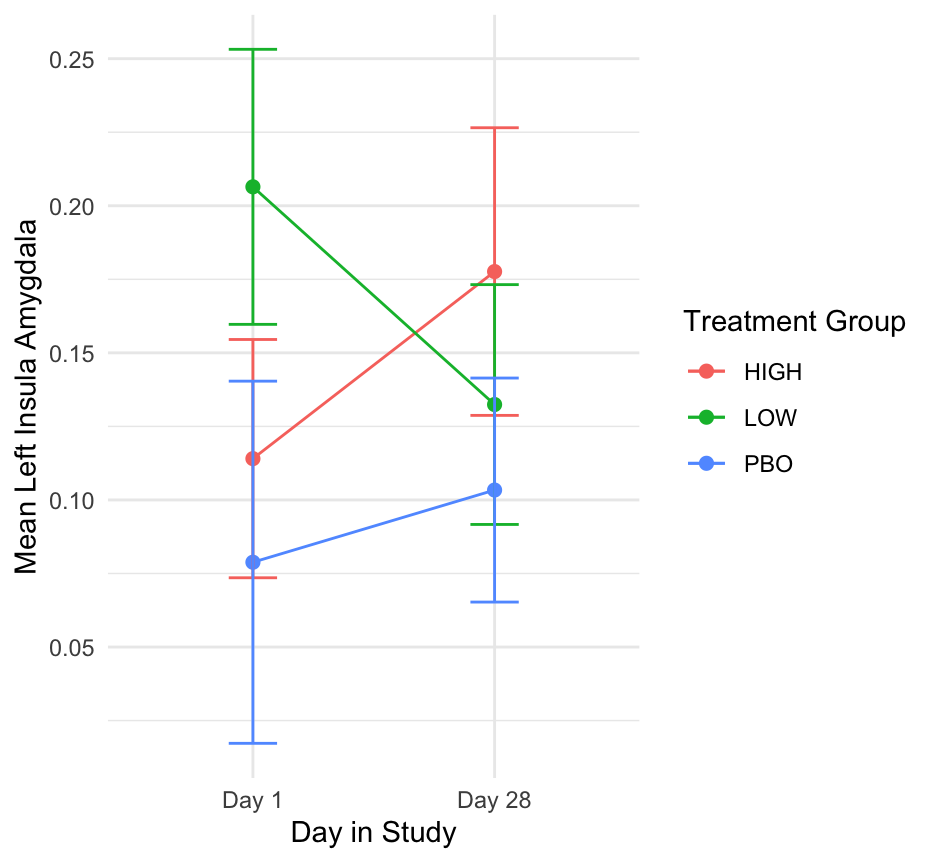
**

*Note:* Mean Left Insula - Amygdala connectivity with standard error bars across study days, by treatment group.

**Figure S8. Mean Right Insula Amygdala**

**
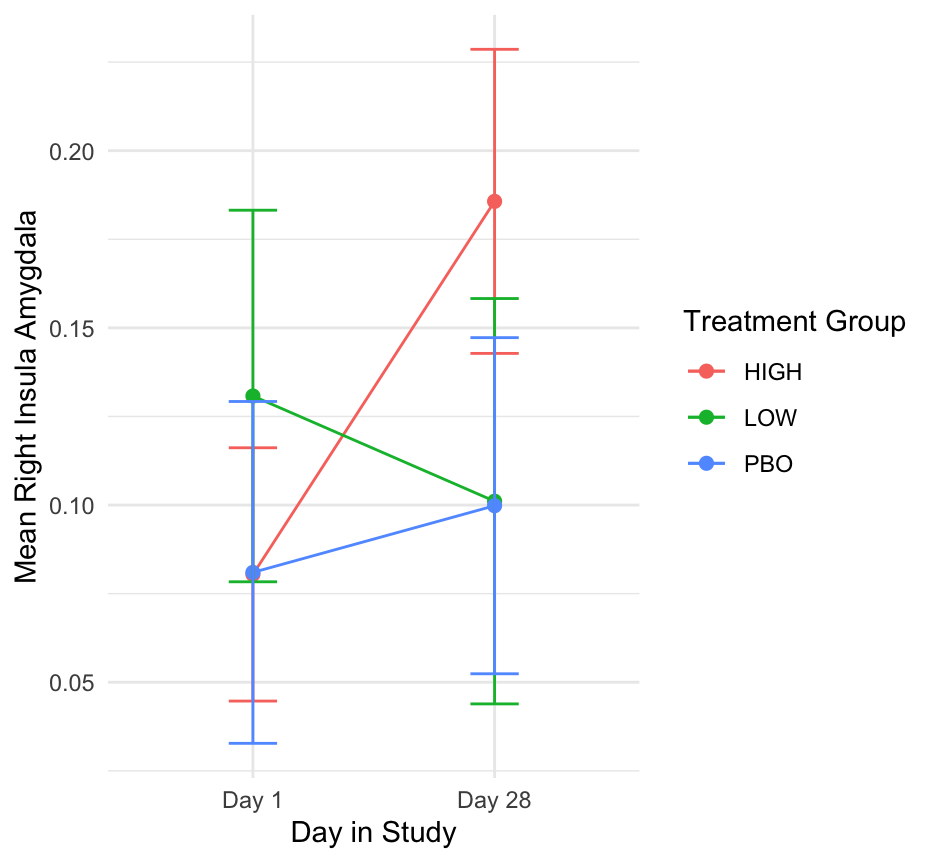
**

*Note:* Mean Right Insula - Amygdala connectivity with standard error bars across study days, by treatment group.

**Figure S9. Mean Insula Amygdala**


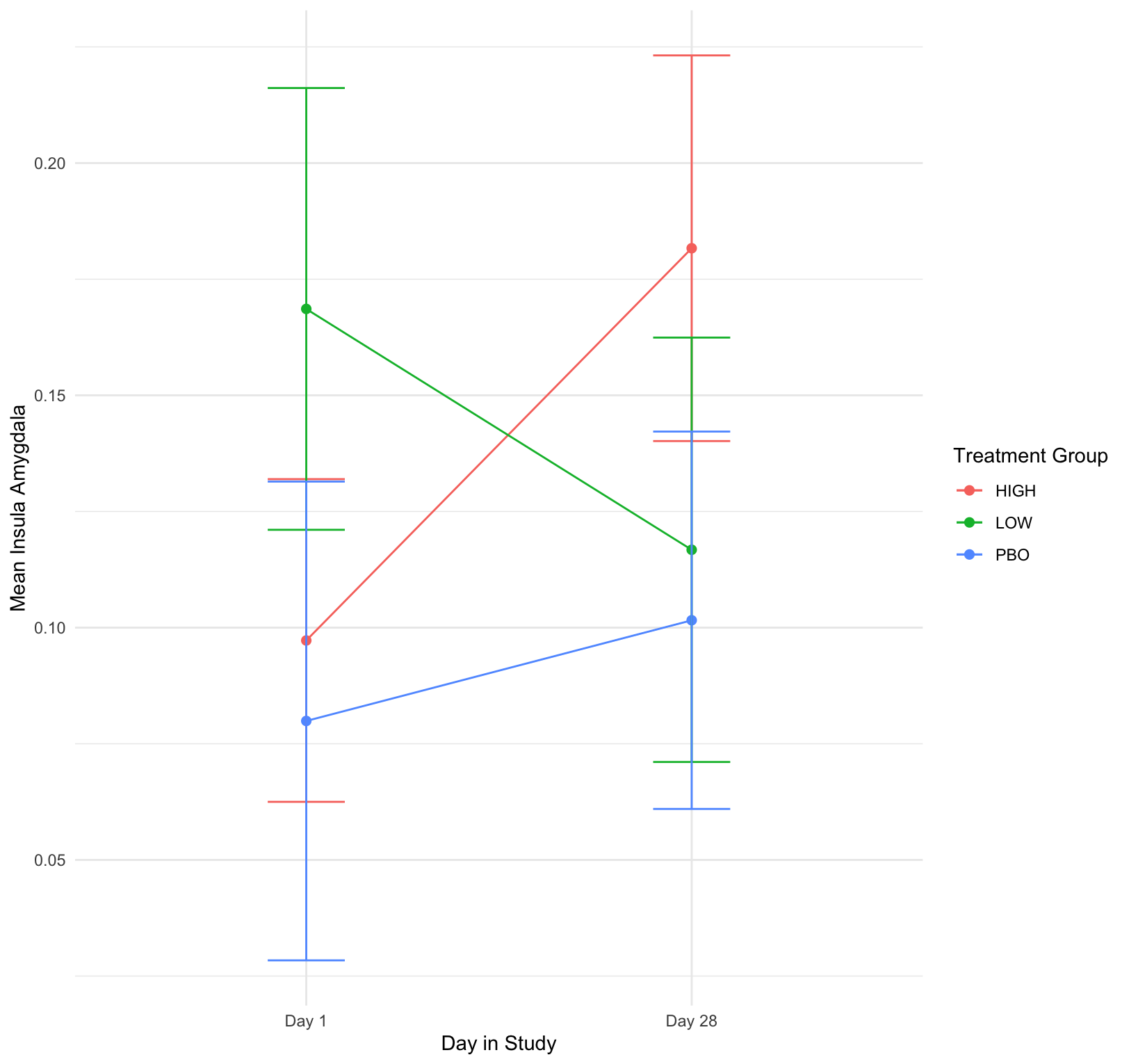


*Note:* Mean Insula - Amygdala connectivity (averaged across hemispheres) with standard error bars across study days, by treatment group.

**Table S4. Between-group Comparisons of Biomarker Differences (Day 28 - Day 1)**

| **Variable** | **Adjusted p-value**  **High vs. Low** | **Adjusted p-value**  **High vs. PBO** | **Adjusted p-value**  **Low vs. PBO** | **Test** |
| --- | --- | --- | --- | --- |
| Difference in blood GSH/GSSG | 0.63 | 0.25 | 0.59 | 1 |
| Difference in brain GSH | 0.84 | 1.00 | 0.96 | 1 |
| % Difference in blood GSH/GSSG | 0.70 | 0.25 | 0.69 | 1 |
| % Difference in brain GSH | 0.81 | 0.99 | 0.92 | 1 |
| Difference in HO-1 | 0.35 | 0.99 | 0.55 | 1 |
| % Difference in HO-1 | 1.00 | 0.99 | 0.99 | 1 |
| Difference in catalase | 0.75 | 1.00 | 0.78 | 1 |
| % Difference in catalase | 0.85 | 0.99 | 0.80 | 1 |
| Steady-state trough NAC levels | 0.54 | NA | NA | 2 |
| pAUC_0-2 | 0.86 | NA | NA | 2 |

*Notes.* 1: Wilcoxon rank sum test with p values adjusted for pairwise comparisons 2: Wilcoxon rank sum test (as PK sample analysis was not conducted for the PBO group, only 2 groups were compared and hence no adjustment to the p-value was needed) Abbreviations: V4, Post-study visit (approximately day 28-32); V2, baseline visit (day 1); GSH, glutathione; GSH/GSSG, redox ratio; HO-1; heme oxygenase; NAC; N-acetyl cysteine; pAUC_0-2, partial area under the curve between 0-2h post dose; NA, not applicable.

**Table S5. Changes in clinical outcomes.**

|  | **Total** | | | **High** | | | **Low** | | | **PBO** | | |
| --- | --- | --- | --- | --- | --- | --- | --- | --- | --- | --- | --- | --- |
| **Measure** | **Baseline Mean (SD)** | **Post Mean (SD)** | ***t* test statistic** | **Baseline Mean (SD)** | **Post Mean (SD)** | ***t* test statistic** | **Baseline Mean (SD)** | **Post Mean (SD)** | ***t* test statistic** | **Baseline Mean (SD)** | **Post Mean (SD)** | ***t* test statistic** |
| BDI-II | 26.81 (9.53) | 21.70 (11.00) | 3.74*** | 30.45 (10.46) | 26.27 (14.72) | 1.77 | 26.29 (8.74) | 20.64 (11.24) | 3.12** | 27.00 (11.43) | 22.14(10.17) | 1.48 |
| BSSI | 6.97 (6.84) | 5.61 (5.39) | 1.45 | 8.45 (6.23) | 7.20 (6.66) | 0.98 | 5.71 (7.53) | 3.21 (5.25) | 2.14 | 8.23 (6.98) | 8.36 (4.53) | -0.45 |
| ABUSI | 13.64 (4.61) | 9.65 (4.77) | 4.67*** | 14.36 (2.80) | 11.27 (6.10) | 1.95 | 12.93 (4.84) | 9.14 (3.28) | 2.53* | 13.23 (5.80) | 8.64 (4.68) | 3.49** |
| PHQ-9 | 12.89 (4.63) | 9.95 (4.87) | 5.14*** | 14.55 (4.55) | 11.55 (6.15) | 2.72* | 12.50 (4.31) | 10.14 (4.62) | 2.62* | 11.31 (4.89) | 8.79 (3.58) | 3.51** |
| ISAS (*ln* total NSSI episodes) | 2.78 (0.63) | 0.48 (0.63) | 10.10*** | 2.27 (2.37) | 0.54 (0.80) | 4.85** | 3.16 (4.82) | 0.97 (1.65) | 7.30*** | 3.12 (2.56) | 0.43 (0.54) | 5.13** |
| ISAS (*ln* total NSSI injuries) | 3.08 (0.45) | 0.48 (0.45) | 4.78*** | 1.76 (1.34) | 0.52 (0.35) | 2.28 | 3.94 (10.87) | 0.61 (0.76) | 2.64* | 3.15 (2.39) | 0.58 (0.47) | 5.74* |

*Note:*  * p < 0.05. ** p < 0.01. *** p < 0.001. BDI-II: Beck Depression Inventory-II. BSSI: Beck Scale for Suicide Ideation. ABUSI: Alexian Brothers Urge to Self-Injure.

PHQ-9: Patient Health Questionnaire-9. ISAS: Inventory of Statements About Self-injury

**Figure S10. PHQ-9**

**
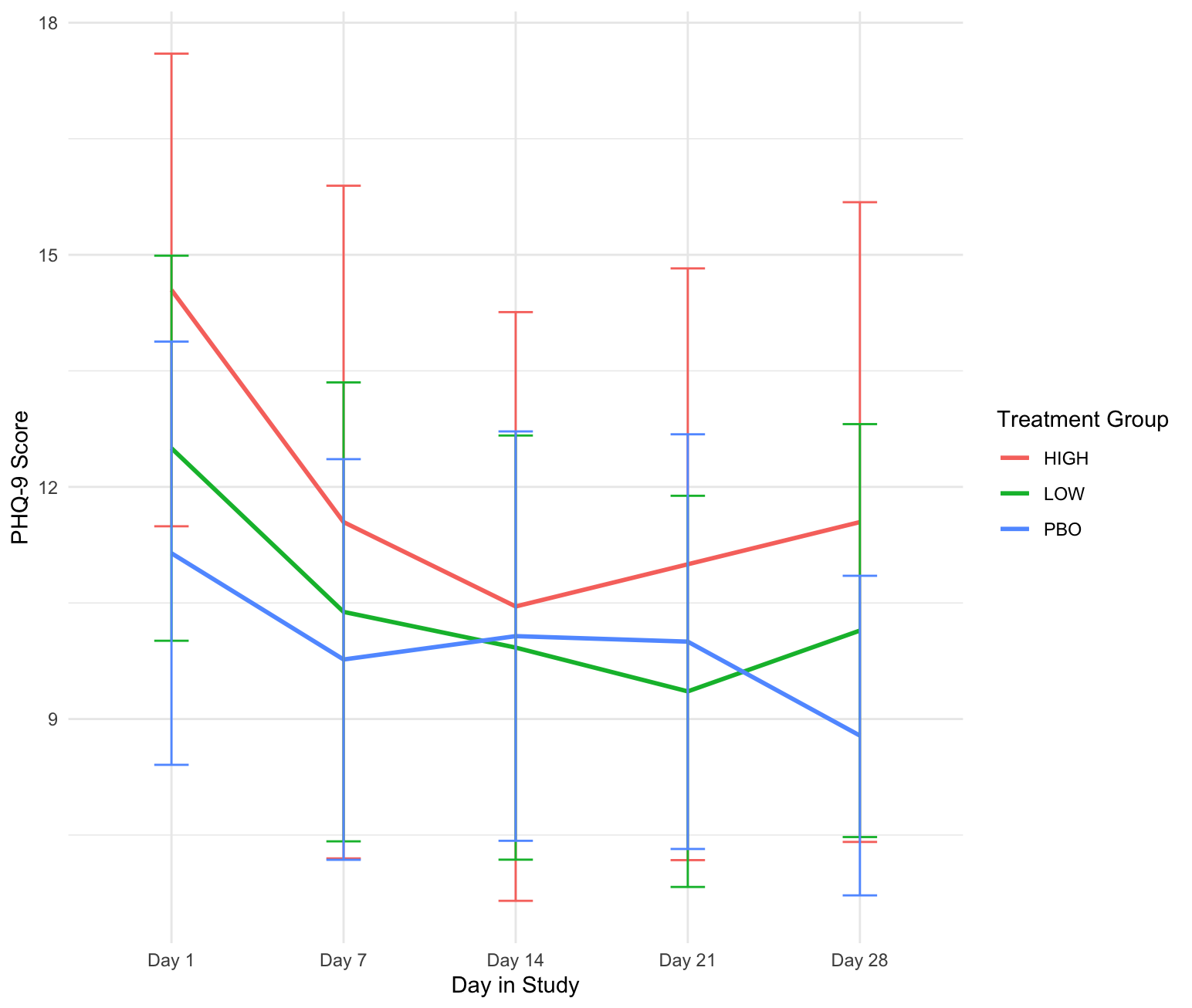
**

*Note:* Mean PHQ-9 scores with standard error bars across study days, by treatment group.

**Figure S11. ISAS episodes**

**
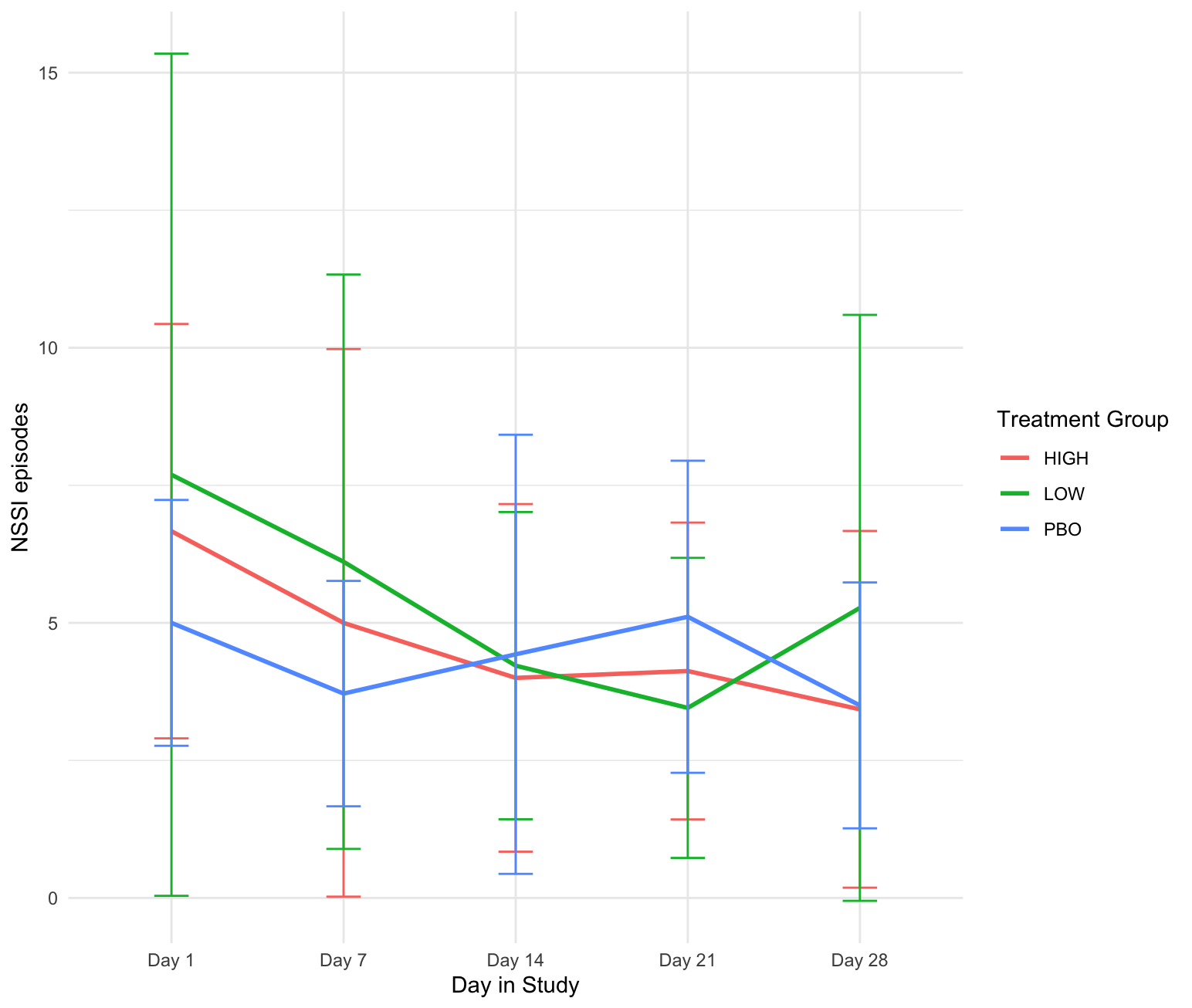
**

*Note:* Mean NSSI episodes with standard error bars across study days, by treatment group.

**Figure S12. ISAS injuries**

**
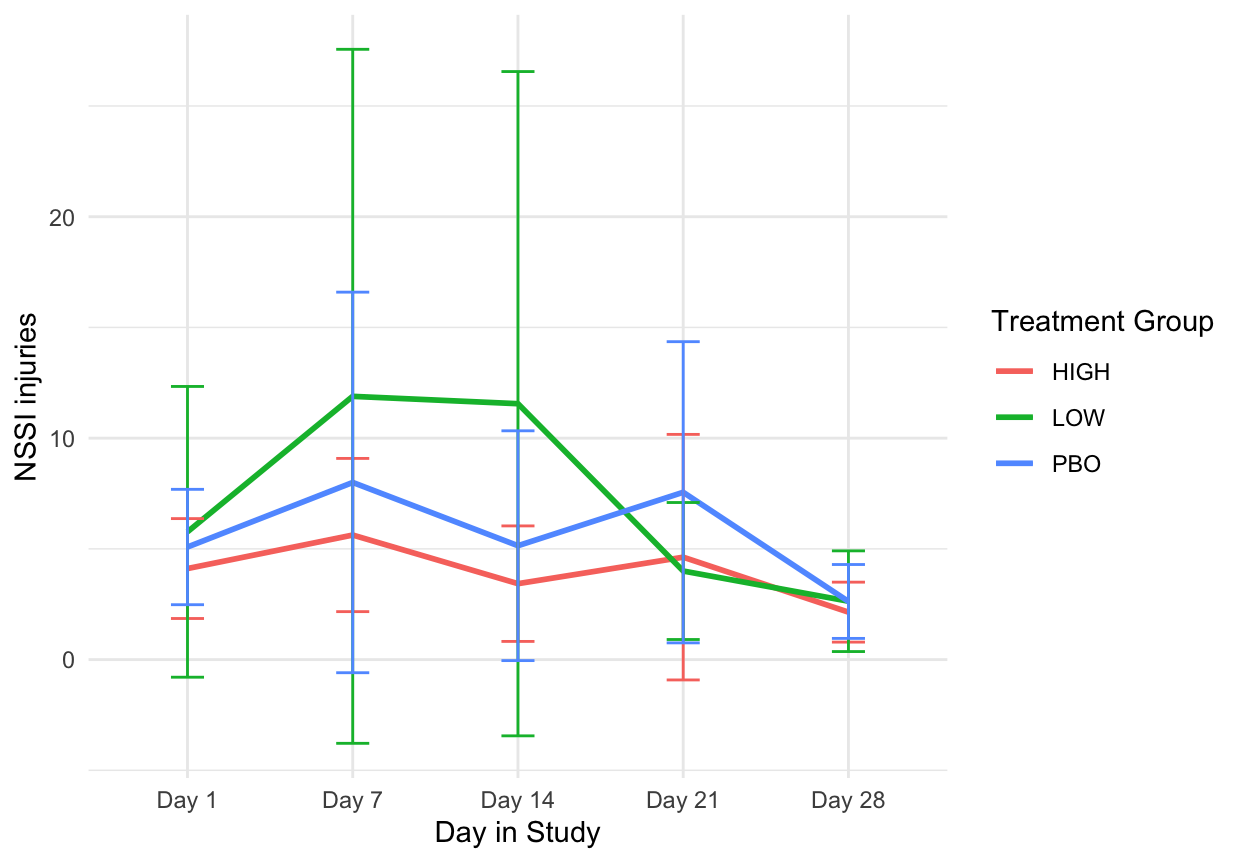
**

*Note:* Mean NSSI injuries with standard error bars across study days, by treatment group.

**Figure S13. BDI-II**

**
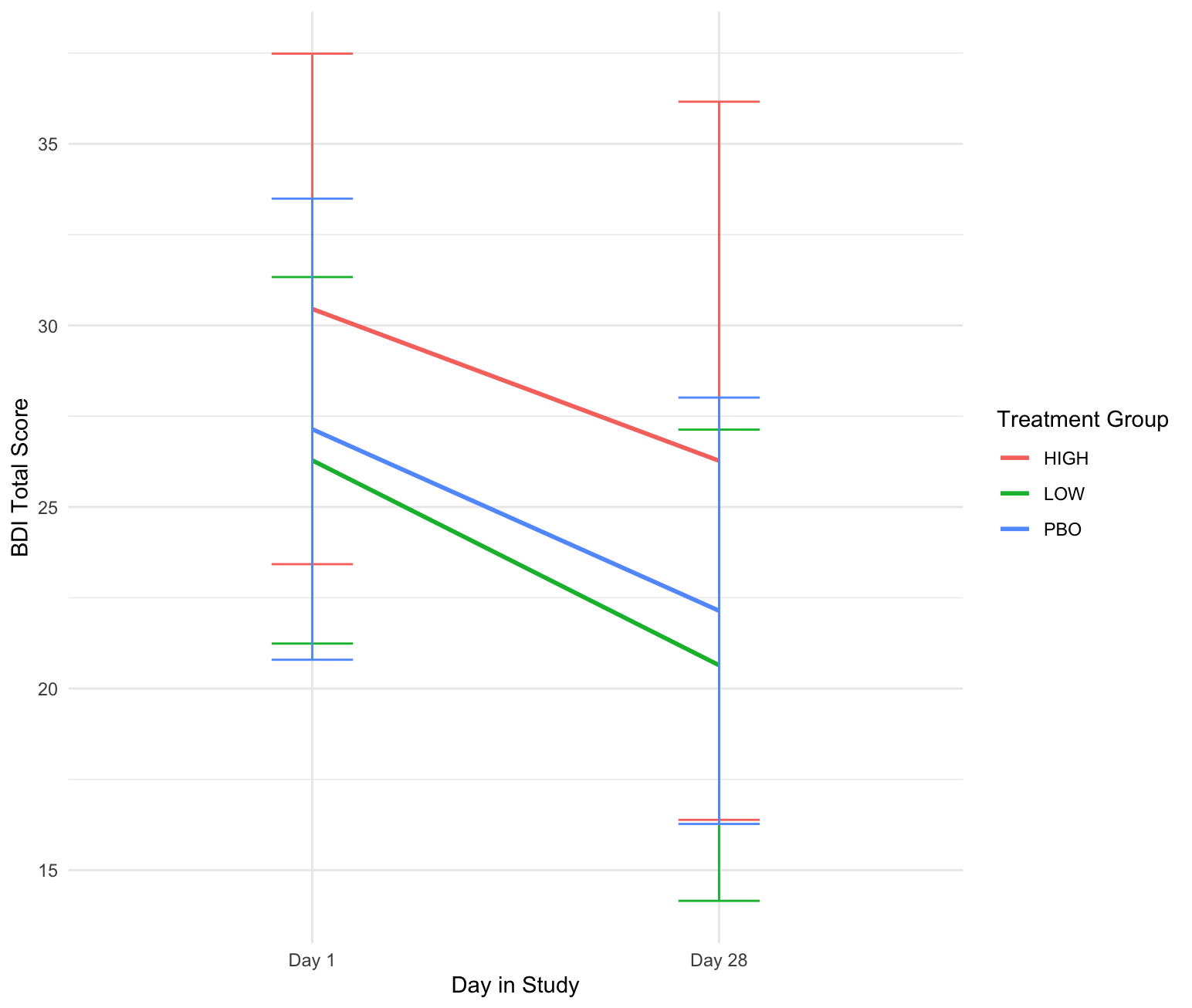
**

*Note:* Mean BDI-II scores with standard error bars across study days, by treatment group.

**Figure S14. BSSI**

**
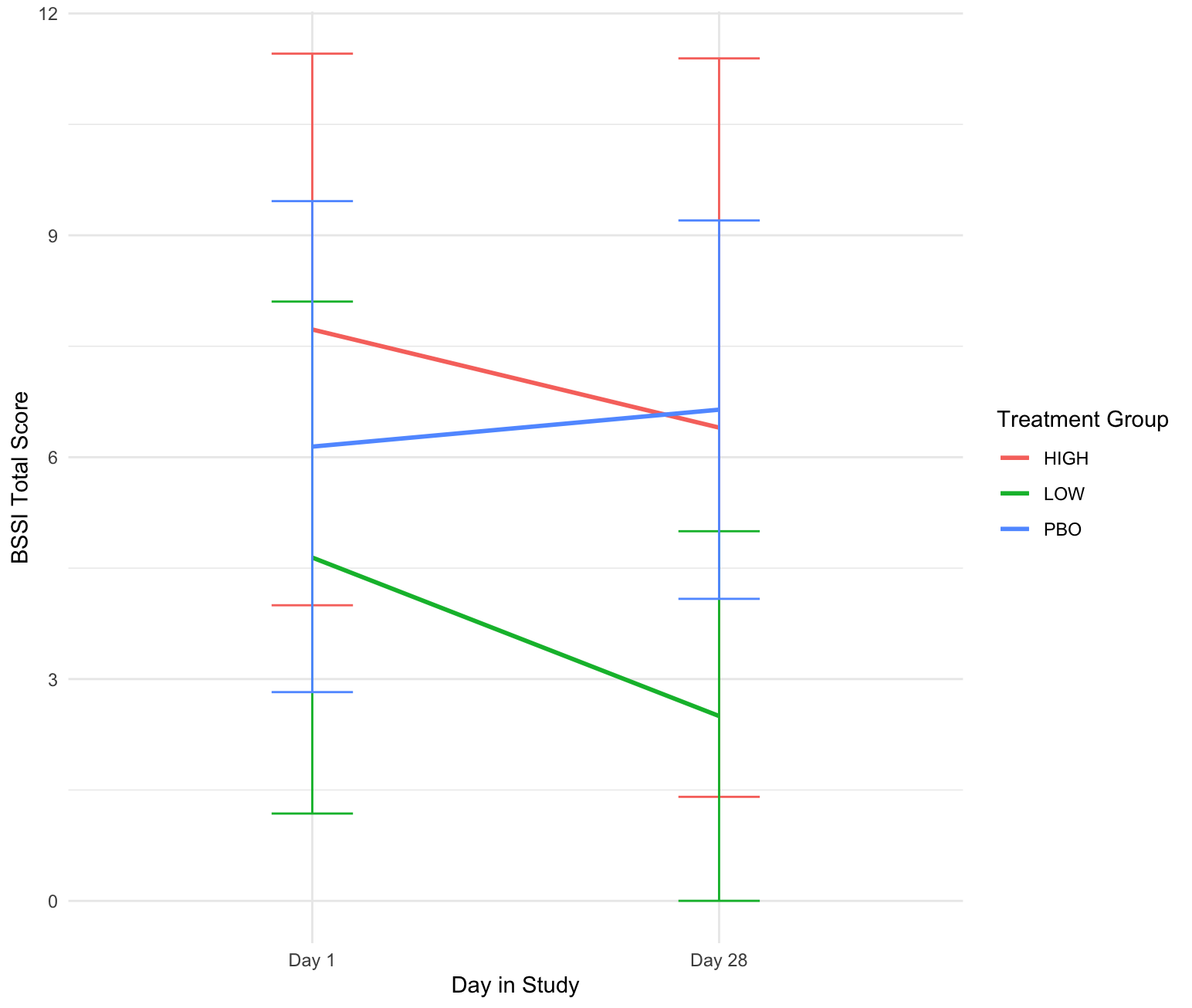
**

*Note:* Mean BSSI scores with standard error bars across study days, by treatment group.

**Figure S15. ABUSI**

**
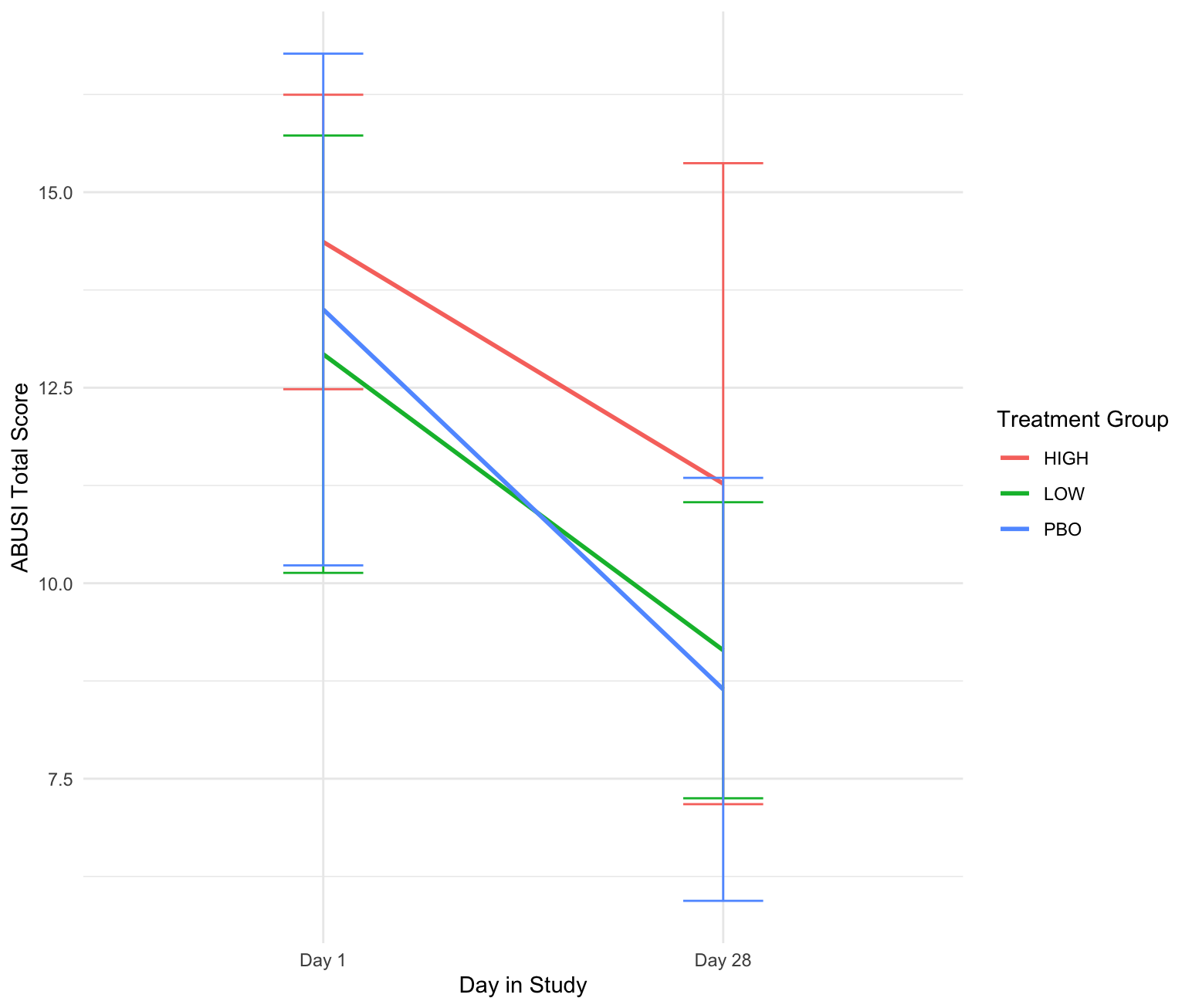
**

*Note:* Mean ABUSI scores with standard error bars across study days, by treatment group.

**Figure S16. Correlation matrix of biological and clinical outcomes in the whole sample (based on N = 43)**

**
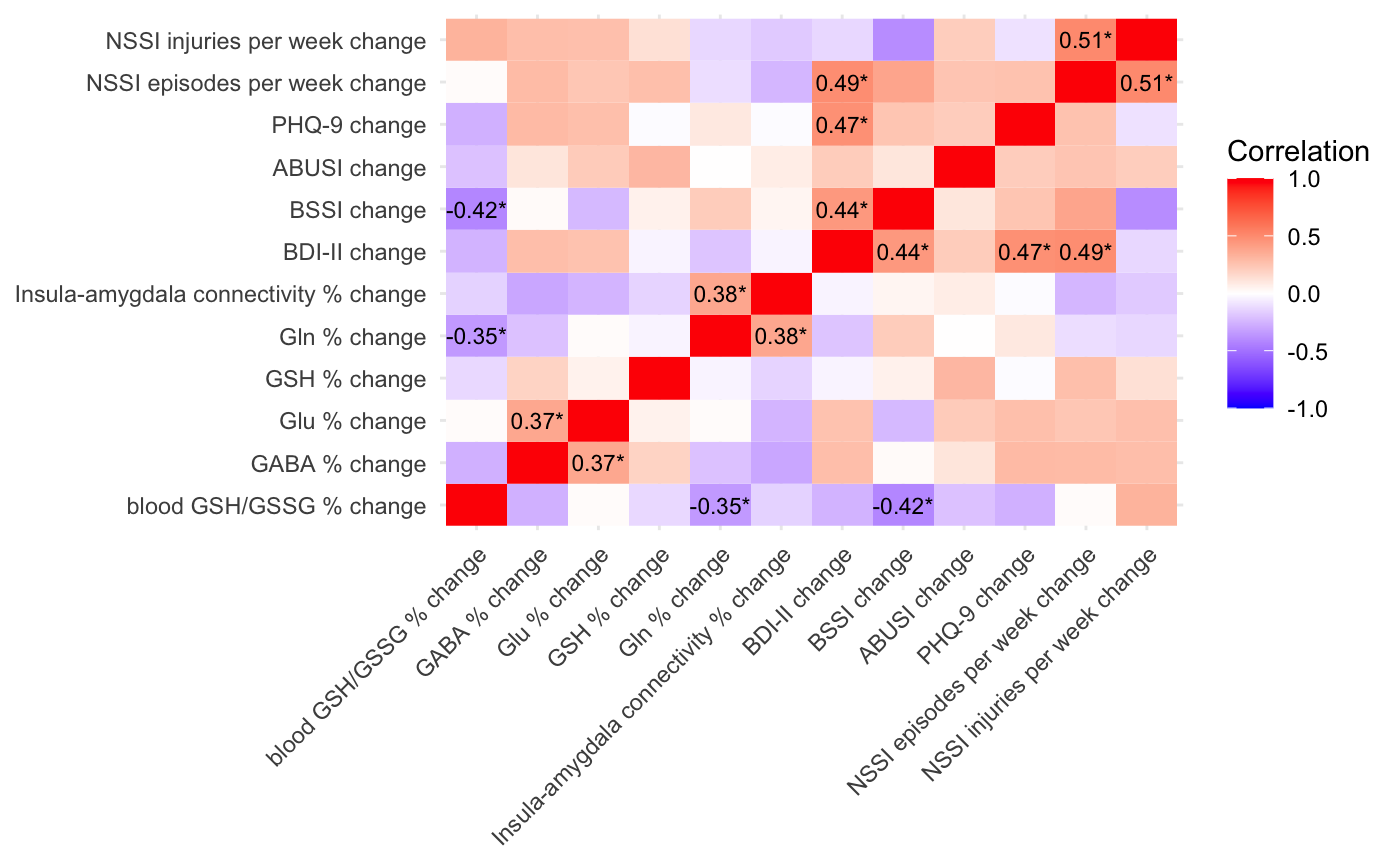
**

*Note:* *unadjusted p < 0.05.

**Figure S17. Correlation matrix of biological and clinical outcomes in the High group (based on N = 13)**

**
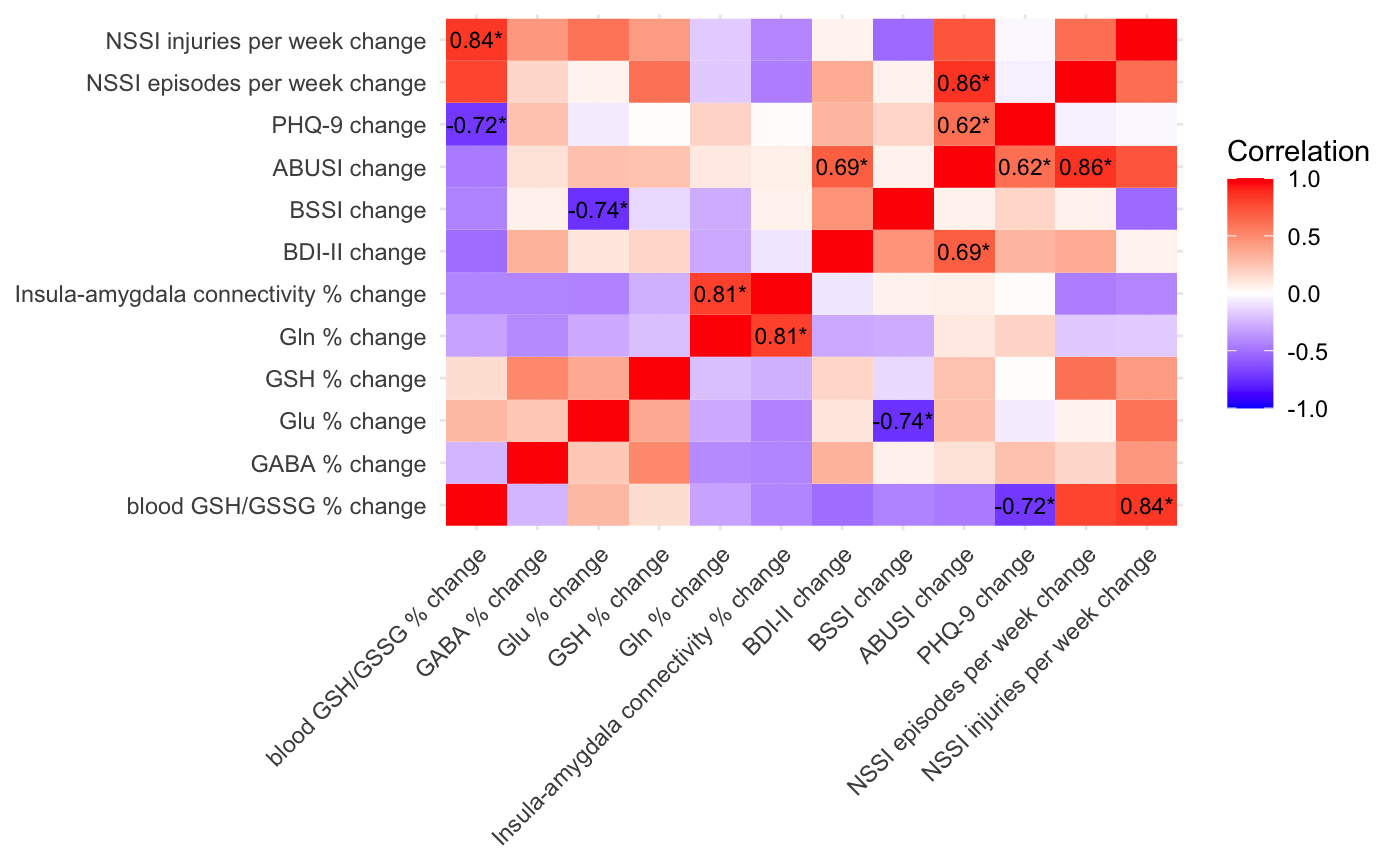
**

*Note:* *unadjusted p < 0.05.

**Figure S18. Correlation matrix of biological and clinical outcomes in the Low group (based on N = 15)**

**
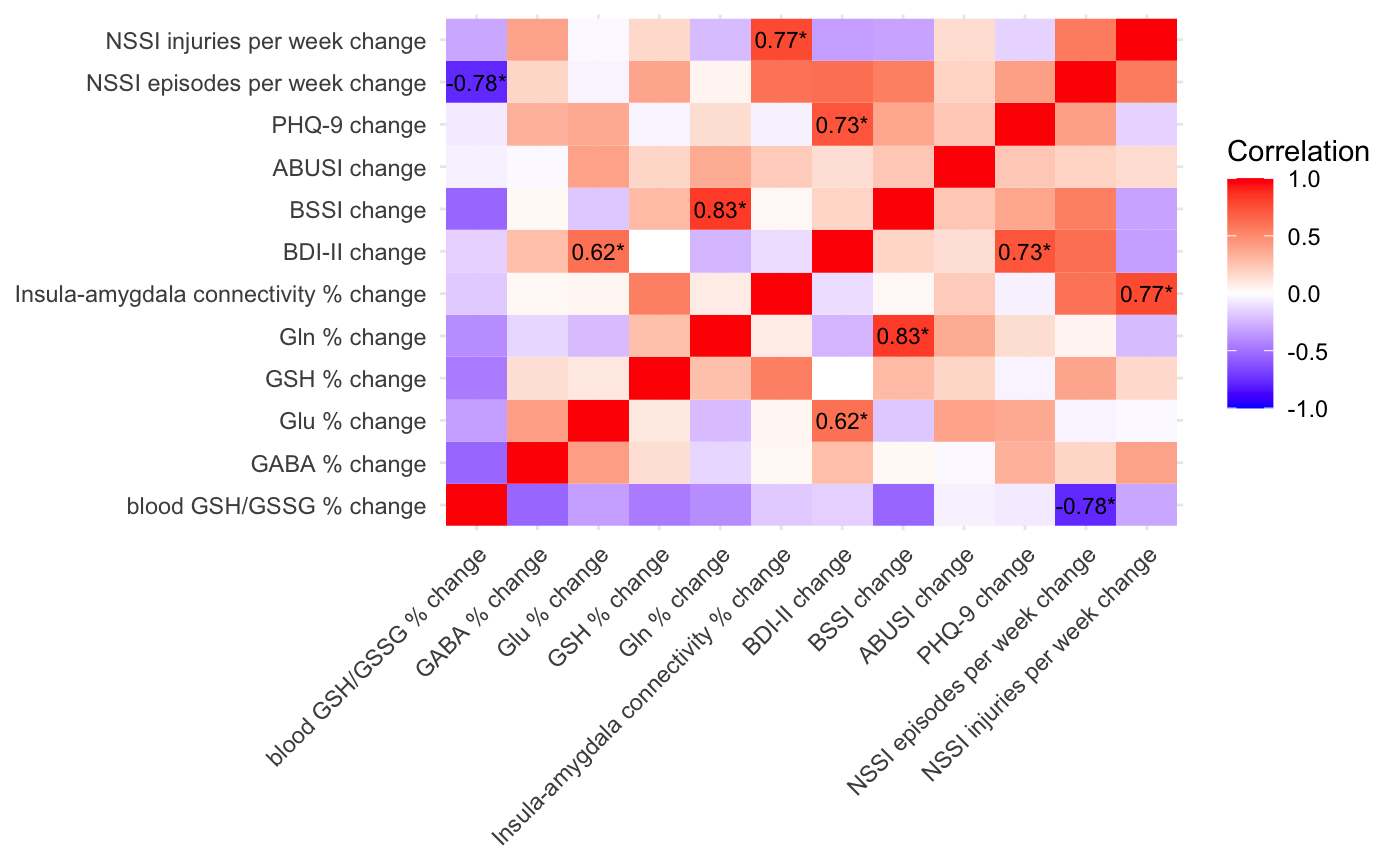
**

*Note:* *unadjusted p < 0.05.

**Figure S19. Correlation matrix of biological and clinical outcomes in the PBO group (based on N = 15)**


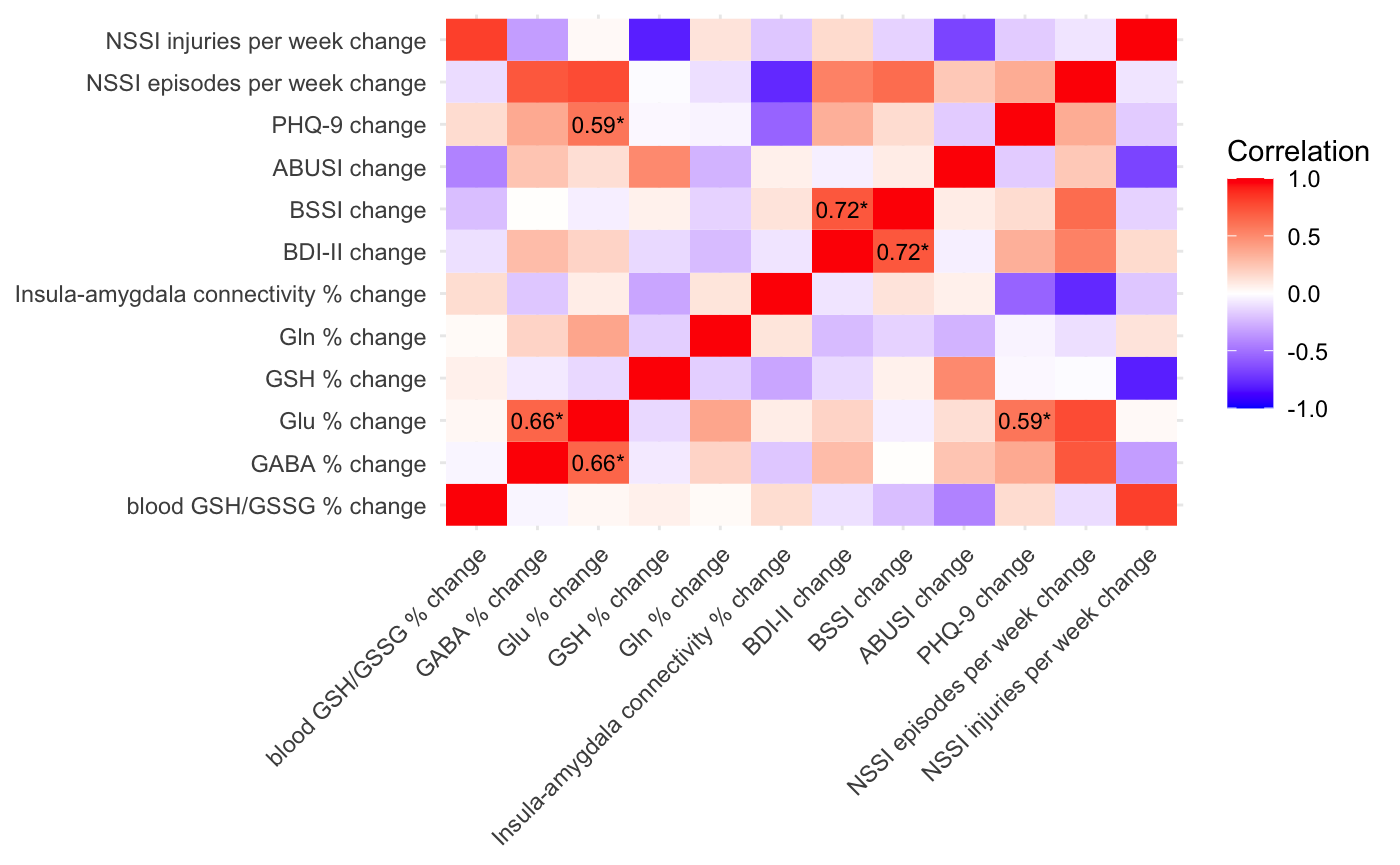


*Note:* *unadjusted p < 0.05.

**Table S6. Condensed Summary Counts of Side Effects per Body System**

Total N enrolled = 43; N = 4 dropped out before Tx dispensed; Table is based on N = 39.

| **Characteristic** | **Day 01**, N = 373 **Day 07**, N = 325 **Day 14**, N = 300 **Day 21**, N = 271 **Day 28**, N = 257 | | | | |
| --- | --- | --- | --- | --- | --- |
| Body Systems |  |  |  |  |  |
| Nervous System | 189 (51%) | 159 (49%) | 149 (50%) | 141 (52%) | 143 (56%) |
| Cardiovascular | 30 (8.0%) | 22 (6.8%) | 25 (8.3%) | 18 (6.6%) | 20 (7.8%) |
| Gastrointestinal | 79 (21%) | 82 (25%) | 72 (24%) | 59 (22%) | 56 (22%) |
| Skin | 17 (4.6%) | 21 (6.5%) | 18 (6.0%) | 11 (4.1%) | 8 (3.1%) |
| Ear Nose Throat | 32 (8.6%) | 24 (7.4%) | 20 (6.7%) | 24 (8.9%) | 19 (7.4%) |
| Genitourinary | 8 (2.1%) | 5 (1.5%) | 5 (1.7%) | 6 (2.2%) | 3 (1.2%) |
| Musculoskeletal | 16 (4.3%) | 12 (3.7%) | 11 (3.7%) | 11 (4.1%) | 8 (3.1%) |
| Other | 2 (0.5%) | 0 (0%) | 0 (0%) | 1 (0.4%) | 0 (0%) |

**Table S7. Expanded Summary of Frequency and Severity of Side Effects**

Total N enrolled = 43; N = 4 dropped out before Tx dispensed; Table is based on N = 39.

| **Characteristic***^^[[1]](#footnote-1)^^* | **Day 01**, N =  373*^1^* | **Day 07**, N =  325*^1^* | **Day 14**, N =  300*^1^* | **Day 21**, N =  271*^1^* | **Day 28**, N =  257*^1^* |
| --- | --- | --- | --- | --- | --- |
| **Nervous System** |  |  |  |  |  |
| Frequency |  |  |  |  |  |
| 1-2 days | 99 (52%) | 95 (60%) | 87 (58%) | 89 (63%) | 81 (57%) |
| 3-4 days | 48 (25%) | 47 (30%) | 35 (23%) | 28 (20%) | 36 (25%) |
| 5-7 days | 42 (22%) | 17 (11%) | 27 (18%) | 24 (17%) | 26 (18%) |
| Missing | 0 (0%) | 0 (0%) | 0 (0%) | 0 (0%) | 0 (0%) |
| Severity |  |  |  |  |  |
| Mild | 85 (45%) | 85 (53%) | 73 (49%) | 78 (55%) | 75 (52%) |
| Moderate | 88 (47%) | 67 (42%) | 65 (44%) | 54 (38%) | 56 (39%) |
| Severe | 14 (7.4%) | 7 (4.4%) | 10 (6.7%) | 7 (5.0%) | 11 (7.7%) |
| Missing | 2 (1.1%) | 0 (0%) | 1 (0.7%) | 2 (1.4%) | 1 (0.7%) |
| **Cardiovascular** |  |  |  |  |  |

| Frequency |  |  |  |  |  |
| --- | --- | --- | --- | --- | --- |
| 1-2 days | 20 (67%) | 16 (73%) | 17 (68%) | 12 (67%) | 14 (70%) |
| 3-4 days | 7 (23%) | 3 (14%) | 5 (20%) | 4 (22%) | 3 (15%) |
| 5-7 days | 3 (10%) | 3 (14%) | 3 (12%) | 2 (11%) | 3 (15%) |
| Missing | 0 (0%) | 0 (0%) | 0 (0%) | 0 (0%) | 0 (0%) |
| Severity |  |  |  |  |  |
| Mild | 18 (60%) | 13 (59%) | 18 (72%) | 8 (44%) | 14 (70%) |
| Moderate | 10 (33%) | 7 (32%) | 6 (24%) | 10 (56%) | 4 (20%) |
| Severe | 2 (6.7%) | 2 (9.1%) | 1 (4.0%) | 0 (0%) | 2 (10%) |
| Missing | 0 (0%) | 0 (0%) | 0 (0%) | 0 (0%) | 0 (0%) |
| **Gastrointestinal** |  |  |  |  |  |
| Frequency |  |  |  |  |  |
| 1-2 days | 53 (67%) | 57 (70%) | 56 (78%) | 44 (75%) | 34 (61%) |
| 3-4 days | 18 (23%) | 21 (26%) | 10 (14%) | 13 (22%) | 19 (34%) |
| 5-7 days | 8 (10%) | 4 (4.9%) | 6 (8.3%) | 2 (3.4%) | 3 (5.4%) |
| Missing | 0 (0%) | 0 (0%) | 0 (0%) | 0 (0%) | 0 (0%) |
| Severity |  |  |  |  |  |
| Mild | 52 (66%) | 55 (67%) | 46 (64%) | 38 (64%) | 30 (54%) |
| Moderate | 25 (32%) | 27 (33%) | 24 (33%) | 19 (32%) | 22 (39%) |
| Severe | 2 (2.5%) | 0 (0%) | 2 (2.8%) | 2 (3.4%) | 4 (7.1%) |
| Missing | 0 (0%) | 0 (0%) | 0 (0%) | 0 (0%) | 0 (0%) |
| **Skin** |  |  |  |  |  |
| Frequency |  |  |  |  |  |
| 1-2 days | 9 (53%) | 14 (67%) | 7 (39%) | 5 (45%) | 4 (50%) |
| 3-4 days | 3 (18%) | 5 (24%) | 4 (22%) | 3 (27%) | 1 (12%) |
| 5-7 days | 5 (29%) | 2 (9.5%) | 7 (39%) | 3 (27%) | 3 (38%) |
| Missing | 0 (0%) | 0 (0%) | 0 (0%) | 0 (0%) | 0 (0%) |
| Severity |  |  |  |  |  |

| Mild | 13 (76%) | 16 (76%) | 13 (72%) | 7 (64%) | | 6 (75%) |
| --- | --- | --- | --- | --- | --- | --- |
| Moderate | 3 (18%) | 5 (24%) | 4 (22%) | 4 (36%) | | 2 (25%) |
| Severe | 1 (5.9%) | 0 (0%) | 1 (5.6%) | 0 (0%) | | 0 (0%) |
| Missing | 0 (0%) | 0 (0%) | 0 (0%) | 0 (0%) | | 0 (0%) |
| **Ear Nose Throat** |  |  |  |  | |  |
| Frequency |  |  |  |  | |  |
| 1-2 days | 20 (62%) | 14 (58%) | 11 (55%) | 12 (50%) | | 15 (79%) |
| 3-4 days | 6 (19%) | 8 (33%) | 6 (30%) | 8 (33%) | | 2 (11%) |
| 5-7 days | 6 (19%) | 2 (8.3%) | 3 (15%) | 4 (17%) | | 2 (11%) |
| Missing | 0 (0%) | 0 (0%) | 0 (0%) | 0 (0%) | | 0 (0%) |
| Severity |  |  |  |  | |  |
| Mild | 29 (91%) | 18 (75%) | 17 (85%) | 20 (83%) | | 15 (79%) |
| Moderate | 3 (9.4%) | 6 (25%) | 3 (15%) | 4 (17%) | | 4 (21%) |
| Severe | 0 (0%) | 0 (0%) | 0 (0%) | 0 (0%) | | 0 (0%) |
| Missing | 0 (0%) | 0 (0%) | 0 (0%) | 0 (0%) | | 0 (0%) |
| **Genitourinary** |  |  |  |  | |  |
| Frequency |  |  |  |  | |  |
| 1-2 days | 5 (62%) | 2 (40%) | 2 (40%) | 4 (67%) | | 2 (67%) |
| 3-4 days | 1 (12%) | 0 (0%) | 3 (60%) | 0 (0%) | | 1 (33%) |
| 5-7 days | 2 (25%) | 3 (60%) | 0 (0%) | 2 (33%) | | 0 (0%) |
| Missing | 0 (0%) | 0 (0%) | 0 (0%) | 0 (0%) | | 0 (0%) |
| Severity |  |  |  |  | |  |
| Mild | 6 (75%) | 3 (60%) | 3 (60%) | 3 (50%) | | 2 (67%) |
| Moderate | 2 (25%) | 2 (40%) | 2 (40%) | 3 (50%) | | 1 (33%) |
| Severe | 0 (0%) | 0 (0%) | 0 (0%) | 0 (0%) | | 0 (0%) |
| Missing | 0 (0%) | 0 (0%) | 0 (0%) | 0 (0%) | | 0 (0%) |
| **Musculoskeletal** |  |  |  |  | |  |
| Frequency |  |  |  |  | |  |
| 1-2 days | 8 (50%) | 7 (58%) | 8 (73%) | 8 (73%) | 4 (50%) | |
| 3-4 days | 5 (31%) | 3 (25%) | 2 (18%) | 2 (18%) | 2 (25%) | |
| 5-7 days | 3 (19%) | 2 (17%) | 1 (9.1%) | 1 (9.1%) | 2 (25%) | |
| Missing | 0 (0%) | 0 (0%) | 0 (0%) | 0 (0%) | 0 (0%) | |
| Severity |  |  |  |  |  | |
| Mild | 9 (56%) | 7 (58%) | 10 (91%) | 10 (91%) | 5 (62%) | |
| Moderate | 7 (44%) | 4 (33%) | 0 (0%) | 1 (9.1%) | 3 (38%) | |
| Severe | 0 (0%) | 0 (0%) | 0 (0%) | 0 (0%) | 0 (0%) | |
| Missing | 0 (0%) | 1 (8.3%) | 1 (9.1%) | 0 (0%) | 0 (0%) | |
| **Other** |  |  |  |  |  | |
| Frequency |  |  |  |  |  | |
| 1-2 days | 0 (0%) | 0 (0%) |  |  |  | |
| 3-4 days | 0 (0%) | 1 (100%) |  |  |  | |
| 5-7 days | 2 (100%) | 0 (0%) |  |  |  | |
| Missing | 0 (0%) | 0 (0%) |  |  |  | |
| Severity |  |  |  |  |  | |
| Mild | 2 (100%) | 0 (0%) |  |  |  | |
| Moderate | 0 (0%) | 1 (100%) |  |  |  | |
| Severe | 0 (0%) | 0 (0%) |  |  |  | |
| Missing | 0 (0%) | 0 (0%) |  |  |  | |

^4^ Number of events (%)

**Supplemental References**

1. [Kartha RV, Zhou J, Basso L, Schröder H, Orchard PJ, Cloyd J (2015) Mechanisms of Antioxidant Induction with High-Dose N-Acetylcysteine in Childhood Cerebral Adrenoleukodystrophy. CNS Drugs 29:1041–1047](http://paperpile.com/b/U88ojx/ZsF1U)

2. [Holmay MJ, Terpstra M, Coles LD, Mishra U, Ahlskog M, Öz G, Cloyd JC, Tuite PJ (2013) N-acetylcysteine boosts brain and blood glutathione in Gaucher and Parkinson diseases. Clin Neuropharmacol 36:103–106](http://paperpile.com/b/U88ojx/q9b6g)

3. [King B, Vance J, Wall GM, Shoup R (2019) Quantitation of free and total N-acetylcysteine amide and its metabolite N-acetylcysteine in human plasma using derivatization and electrospray LC-MS/MS. Journal of Chromatography B 1109:25–36](http://paperpile.com/b/U88ojx/LbQmj)

4. [Wheeler OR (1990) Salzman JA, Elsayed NM, Omaye ST. Korte DW Jr. Automated assays for superoxide dismutase, catalase, glutathione peroxidase, and glutathione …. Anal. Biochem.](http://paperpile.com/b/U88ojx/Sjgii)

5. [Johanson LH, Borg HLA (1988) A spectrophotometric method for determination of catalase activity in small tissue sample. Anal. Biochem.](http://paperpile.com/b/U88ojx/CcjgC)

6. [Kartha RV, Terluk MR, Brown R, Travis A, Mishra UR, Rudser K, Lau H, Jarnes JR, Cloyd JC, Weinreb NJ (2020) Patients with Gaucher disease display systemic oxidative stress dependent on therapy status. Mol Genet Metab Rep 25:100667](http://paperpile.com/b/U88ojx/HsN3t)

1. Number of events (%) [↑](#footnote-ref-1)
